# Mutual population-shift driven antibody-peptide binding elucidated by molecular dynamics simulations

**DOI:** 10.1038/s41598-020-58320-z

**Published:** 2020-01-29

**Authors:** Gert-Jan Bekker, Ikuo Fukuda, Junichi Higo, Narutoshi Kamiya

**Affiliations:** 1 Institute for Protein Research, Osaka University, 3-2 Yamadaoka, Suita, Osaka 565-0871 Japan; 2 Graduate School of Simulation Studies, University of Hyogo, 7-1-28 Minatojima Minami-machi, Chuo-ku, Kobe, Hyogo 650-0047 Japan

**Keywords:** Computational biophysics, Biophysical chemistry, Computational chemistry

## Abstract

Antibody based bio-molecular drugs are an exciting, new avenue of drug development as an alternative to the more traditional small chemical compounds. However, the binding mechanism and the effect on the conformational ensembles of a therapeutic antibody to its peptide or protein antigen have not yet been well studied. We have utilized dynamic docking and path sampling simulations based on all-atom molecular dynamics to study the binding mechanism between the antibody solanezumab and the peptide amyloid-β (Aβ). Our docking simulations reproduced the experimental structure and gave us representative binding pathways, from which we accurately estimated the binding free energy. Not only do our results show why solanezumab has an explicit preference to bind to the monomeric form of Aβ, but that upon binding, both molecules are stabilized towards a specific conformation, suggesting that their complex formation follows a novel, mutual population-shift model, where upon binding, both molecules impact the dynamics of their reciprocal one.

## Introduction

With pathogen targeting antibodies being one of the key treatments in immunotherapy^1,2^, molecular recognition of an antibody to its antigen proteins or peptides has attracted great attention^3^. Understanding the recognition mechanism provides crucial information for rational design of an antibody^4^, which could potentially be attained by studying the native binding configuration at atomistic resolution. Although X-ray crystallography is a powerful tool, the binding mechanism cannot be elucidated, as it is not possible to investigate the binding process via intermediary structures. In contrast, while molecular dynamics (MD) simulations offer the possibility to investigate the binding mechanism by studying the entire conformational space, they cannot reach the timescales that biologically interesting phenomena occur at. Enhanced MD simulation techniques such as multicanonical MD (McMD)^5–14^ accelerate the dynamics while having the innate ability to re-obtain the canonical ensemble at a given temperature. This enables us to study phenomena such as the binding between molecules at atomistic resolution, not offered by either experimental methods or conventional MD simulations. Recently, docking using advanced MD, such as McMD, Replica Exchange MD^15^, Filling Potential^16^, Metadynamics^17^, Accelerated MD^18^ and even Markov State Modelling of MD simulations^19^, has attracted much attention in the field of protein-drug interaction, where such methods have been coined dynamic docking^20^.

Currently, three basic models exist for the molecular recognition by antibodies; lock-and-key, conformational selection and induced fit^21–26^. In the lock-and-key model, no conformational change of the antibody is required for the antigen to bind. On the other hand, for the conformational selection model, also known as the population-shift model, the two molecules only bind when the antibody is in a bound-like conformation before attempting to bind, leading to a population-shift. Finally, in the induced fit model, binding is attempted while the antibody is in a non-native state, and during the binding process they slowly adapt to each other to eventually reach the native bound state. While antibodies complexed with small compounds as their antigen have been relatively well studied^21–23,25,26^, studies between antibodies and peptides or proteins^24^ are still rare. Therefore, only the antibody structure is considered when assigning a recognition mechanism. Here we strive to apply our established methods^13,14^ to the still greater challenging biological system of antibody-peptide binding, in order to investigate their binding mechanism.

Anti-Alzheimer’s disease drugs have been a raging topic for the past couple of years with several promising antibody drugs developed^3,27^. Recently however, several of these antibody drugs against Amyloid-β (Aβ) that were in clinical trials have been pulled, citing causes such as low efficacy *in vivo*^28^. It’s clear that our basic understanding of the root cause of Alzheimer’s disease isn’t sufficient, and some groups have even moved their focus away from an Aβ-oriented approach^29^. On the other hand, because our understanding of how antibodies interact with highly flexible molecules such as peptides is also lacking, it prevents us from designing more effective antibodies. Given the high, pM-order affinity of the antibody solanezumab to specifically the monomeric form of the Aβ peptide^30^, it would serve as an interesting use-case to investigate their binding mechanism. To this end, we have applied dynamic docking simulations between solanezumab and its Aβ epitope, followed by path sampling simulations to sample the interactions in greater detail to estimate and explain their affinity.

## Results

### Dynamic docking of the Aβ peptide to the antibody

**Figure 1:**
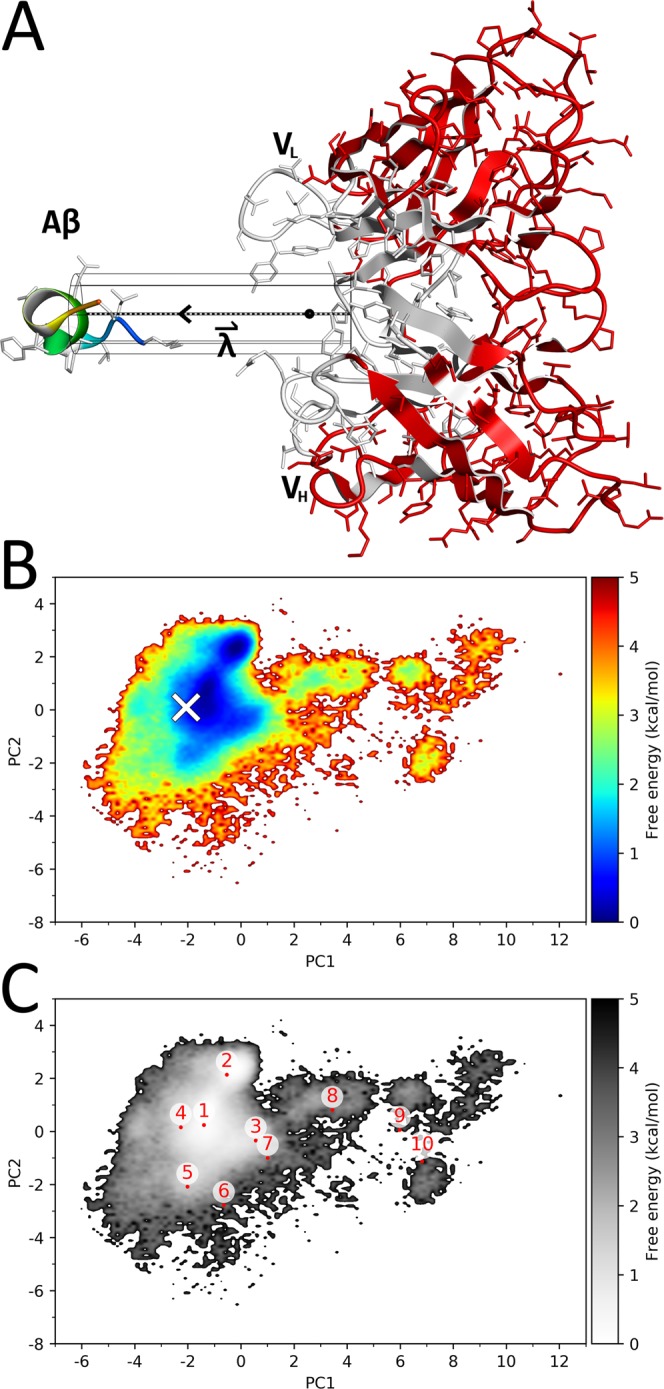
Dynamic docking by McMD. (**A**) The initial starting configuration, which was generated by a high temperature MD simulation with restraints, where the COM of the fragment peptide of Aβ (Lys16-Ser26) was restrained to stay inside the black cylinder (see Methods) and the antibody’s red colored heavy atoms were weakly position restrained. The cylinder ranges from 2.5 Å to 30 Å, where the experimental structure has a λ value of 6.64, indicated by the black dot on the axis of $\mathop{{\rm{\lambda }}}\limits^{\rightharpoonup }$. The sequence numbering of the CDR loops and Aβ are listed in Table S1. The image was drawn using Molmil^46–48^. (**B**) FEL of the ensemble at 300 K projected onto PC1 and PC2, where the white cross marks the experimental structure. See supplementary Section S2 for additional details. (**C**) FEL in greyscale with the locations of the structures **r**_k_ indicated in red.

Dynamic docking starting from the unbound state with the peptide’s center of mass (COM) restrained to stay within a cylinder defined by the axis $\mathop{{\rm{\lambda }}}\limits^{\rightharpoonup }$ (Fig. 1A) was performed, with the simulation details described in the Methods section and supplementary Section S1. The initial structure for the docking simulations is shown in Fig. 1A, which was obtained after approximately 6.3 ns of high temperature MD. After 32 subsequent iterative pre-runs of McMD simulations, we had estimated the density of states, lasting for 17.6 μs (24 × 732.4 ns) in total. During the final production run that lasted for another 24 μs (24 × 1 μs), configurations including bound, unbound and intermediary states were sampled. The flat potential energy distribution from the production run is shown in Fig. S1, which covers a wide temperature region including 300 K, 500 K and 700 K. Fig. 1B shows the free energy landscape (FEL) at room temperature between the Aβ peptide and the antibody along the first and second principal components (PC1 and PC2, respectively). The distribution of the reweighted canonical ensemble at room temperature is narrow in comparison to the wide conformational space of the multicanonical ensemble (Fig. S2). The experimental structure (the white cross in Fig. 1B) is located inside the largest basin, near its global minimum. Combined, these results suggest that our McMD simulations sampled a wide conformational space, including the native state. To analyze the structural ensemble at 300 K, representative structures were obtained, whose locations on the FEL are shown in Fig. 1C as red dots, with the most stable structure, **r**_1_, located near the experimental one. Table 1 lists characteristics regarding each picked representative configuration **r**_k_ as well as the experimental structure, including the root-mean-square deviation (RMSD) and the R(native)-value (see supplementary Section S2 for a detailed description), with the corresponding structures shown in Fig. S3. Compared to the experimental structure, **r**_1_ forms a similar complex structure in terms of atomistic contacts, with an R**(**native)-value of 0.924, while the peptide has an RMSD of 1.83 Å, indicating that our simulations have successfully reproduced the experimental structure. The configurations of **r**_1_, …, **r**_6_ are located within the large basin of the landscape (Fig. 1C) and are structurally very similar in terms of the position of the N-terminal region of the Aβ peptide (panels 1–6 in Fig. S3). Here, the Aβs’ central Phe19-Phe20 residues are buried inside the antibody, as observed in the experimental structure, while only Phe19 is buried in **r**_7_ and **r**_8_ (panels 7 and 8 in Fig. S3). The structures of **r**_9_ and **r**_10_ (panels 9 and 10 in Fig. S3), which are located at the smaller basins of the landscape, differ from those of **r**_1_, …, **r**_8,_ as the peptide binds in an opposite orientation with respect to the first group.

**Table 1:** Characteristics of binding configurations.

struc. id	Cluster free energy	PC1	PC2	PC3	PCA free energy	RASA	R(native)-value	RMSD	λ
**r**_1_	0.00	−1.40	0.25	0.25	0.04	0.38	0.924	1.83	6.75
**r**_2_	0.36	−0.53	2.14	2.14	0.33	0.40	0.828	6.25	5.45
**r**_3_	0.90	0.56	−0.34	−0.34	1.07	0.50	0.504	6.50	7.88
**r**_4_	1.14	−2.27	0.16	0.16	0.90	0.37	0.587	6.37	5.98
**r**_5_	1.41	−2.02	−2.08	−2.08	1.54	0.44	0.925	3.36	6.81
**r**_6_	1.83	−0.65	−2.77	−2.77	4.42	0.49	0.428	5.87	8.75
**r**_7_	2.19	1.01	−0.99	−0.99	2.63	0.47	0.252	9.91	8.01
**r**_8_	2.21	3.44	0.81	0.81	3.29	0.38	0.250	10.78	6.66
**r**_9_	2.55	5.98	0.06	0.06	12.34	0.50	0.065	11.77	8.52
**r**_10_	3.13	6.83	−1.13	−1.13	7.06	0.40	0.131	10.70	7.48
**q**_1_	—	−1.19	0.18	0.06	0.06	0.42	0.961	3.50	6.96
**q**_2_	—	−0.58	2.72	−1.91	0.75	0.36	0.837	6.08	5.34
**q**_3_	—	−0.62	−0.26	0.87	0.51	0.48	0.528	6.37	7.71
**q**_4_	—	−2.78	0.49	−0.94	1.57	0.38	0.590	6.36	5.60
**q**_5_	—	−2.31	−0.88	4.58	1.55	0.39	0.943	3.67	6.85
**q**_6_	—	−1.19	0.42	−0.61	0.05	0.48	0.464	5.91	7.59
**q**_7_	—	1.73	1.04	−4.29	3.69	0.44	0.251	10.11	7.02
**q**_8_	—	1.77	1.44	−2.12	3.39	0.40	0.221	10.48	6.94
**q**_9_	—	8.13	0.91	2.82	5.15	0.52	0.046	12.08	9.11
**q**_10_	—	6.45	−1.91	8.10	7.71	0.46	0.133	10.71	8.22
exp.	—	−2.07	0.09	0.61	0.62	0.36	1.000	0.00	6.64

For each representative structure **r**_k_ (from McMD) and each equilibrated structure **q**_k_ (from refinement MD at 300 K), various characteristics are shown. The relative free energy value in kcal/mol of the corresponding cluster *k* is shown for the structures **r**_k_. For the structures **r**_k_ and **q**_k_, the first three principal components (PC1–3), the free energy in kcal/mol of the point (PC1, PC2) on the FEL in Fig. 1B, the relative accessible surface area (RASA) of Aβ in percentage, the R(native)-value and RMSD in Å with respect to the experimental structure and the λ values are listed. The structures **r**_k_ are ordered by the relative free energy values of their corresponding cluster.

In order to further investigate the stability of the predicted structures **r**_k_, we performed 100 ns canonical MD simulations at 300 K and 400 K without restraints. To quantify the stability, we measured the change in contacts between the peptide and the antibody along the trajectories by calculating the R-value^14,31^, as shown in Fig. S4. These R-values represent the change in intermolecular contacts with respect to each initial structure **r**_k_, where stable binding configurations are expected to maintain their contacts. The trajectories at 300 K show that all structures appear to be stable, with their R-value dropping minimally (see Table S3), illustrating that even non-native structures appear stable at room temperature for simulation lengths accessible by conventional MD simulations. Since the configurations **r**_1_, …, **r**_8_ are structurally very similar in terms of the N-terminal region of the Aβ peptide, it is to be expected that they would behave similarly. Furthermore, the large contact surface between the two molecules enables many contacts to be formed, preventing the molecules from unbinding during our relatively short canonical simulations at room temperature. From these trajectories, representative structures **q**_k_ were obtained by taking the nearest-to-average structure over the final 40 ns, with the resulting structures **q**_k_ shown in Fig. S5, comparing them to their initial structures **r**_k_. In particular, the structures **q**_1_, …, **q**_6_, which are located in the large basin, are very similar to their initial structures. At 400 K however, most binding configurations destabilize, while **r**_1_ and **r**_2_ remain stable even at 400 K, where **r**_2_ is surprisingly stable (R(2) in Fig. S4). This is because the C-terminal side is facing inwards into solanezumab, forming stable contacts (panel 2 in Fig. S3), instead of outwards towards the bulk like **r**_1_ and the experimental structure (panel 1 in Fig. S3). However, considering the full, 42-residue sequence of Aβ, the structure **r**_2_ is unrealistic, as it doesn’t allow for enough space for the C-terminal region to connect to the remainder of the peptide. Besides **r**_2_, the configurations **r**_3_, **r**_4_, **r**_9_ and potentially **r**_10_ where this effect is only partially observed, also have an internally facing C-terminal region. Furthermore, **r**_10_ and potentially **r**_7_ and **r**_8_ where the effect is only partially observed, have an internally facing N-terminal region. Since the inward facing termini would not exist in case of the full-length sequence of Aβ, they could effectively be ignored. Considering the structural incompatibilities of many configurations, as well as their otherwise high structural similarity to **r**_1_, our predicted binding configuration from our dynamic docking, **r**_1_, can be considered to be the most stable structure.

### Path sampling of the Aβ peptide to the antibody

**Figure 2:**
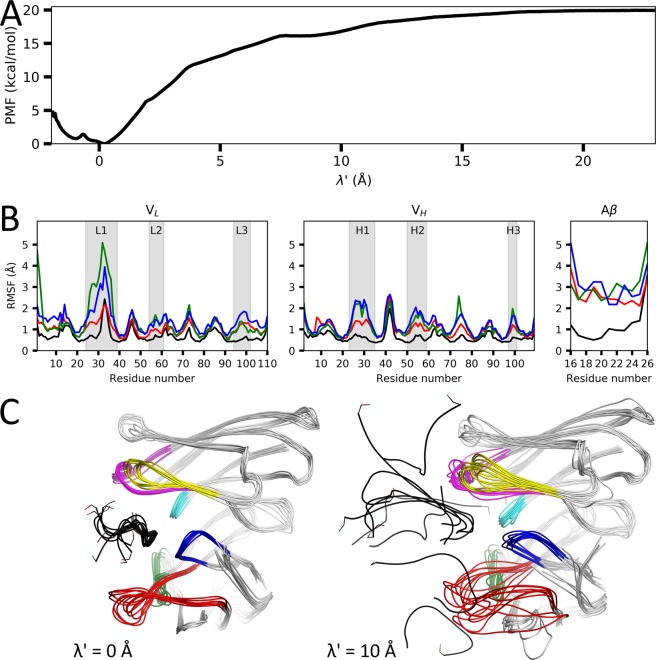
Path sampling by umbrella sampling simulations. (**A**) PMF obtained after applying WHAM, giving a binding affinity of Δ*G* = −19.92 kcal/mol (±0.03). Here, λ′ relates to λ as follows; λ′ = λ − 6.96 Å, where 6.96 Å corresponds to the λ value of the structure **q**_1_. (**B**) RMSF plots of the antibody’s V_L_ and V_H_ (see Methods), and Aβ. Shown are the graphs for the ensembles at λ′ = 0, 5, 10 and 20 Å in black, red, green and blue, respectively. The CDR loops (see Table S1) are indicated and highlighted in grey. (**C**) Random structures picked from the ensemble at λ′ = 0 and 10 Å on the left and right, respectively. The CDR loops L1, L2, L3, H1, H2 and H3 are colored in red, green, blue, magenta, yellow and cyan, respectively and the Aβ peptide is colored in black, with its side-chain of the C-terminal Ser26 shown as a stick model.

Using our previously established method^14^, which is summarized in supplementary Section S3, we estimated representative binding/unbinding pathways from the dynamic docking ensemble starting from the equilibrated structure in the bound state, and then executed path sampling simulations followed by Weighted Histogram Analysis Method (WHAM)^32^ to calculate the binding affinity. This two-staged approach enables us to perform efficient global docking using McMD and then study the binding pathway in-depth using path sampling simulations by focusing on the local interactions and dynamics^13,14^. The picked structures that represent the binding pathway were chosen in such a way that the contact matrix of the interface only slowly changes along λ′ where possible, in order to maximize the structural overlap between windows. For Aβ, this resulted in the N-terminal facing towards the antibody for every picked structure as shown in Fig. S6. We executed a total of 19.2 μs (2 pathways × 16 windows × 3 structures × 200 ns) of Umbrella Sampling (US) simulations at 300 K without position restraints starting from the by the dynamic docking identified representative structures for each pathway derived from **q**_1_ and **q**_2_ to sample the interactions between Aβ and the antibody, which is described in greater detail in Section S4. We then calculated the binding affinity and the error in our calculation using WHAM for both binding configurations. Tables S5 and S6 list the free energy values using different ranges of the trajectories for **q**_1_ and **q**_2_, respectively. For all subsequent analyses, we used the final 100 ns of the production run, where the error ε is the lowest for both **q**_1_ and **q**_2_, after allowing some time for equilibration. The potential of mean force (PMF) of **q**_1_ corresponding to this range is shown in Fig. 2A. Taking the average value over the final 2.5 Å along λ′ (λ value relative to **q**_1_), we obtain binding affinities of Δ*G* = −19.92 kcal/mol (±0.03) and Δ*G* = −19.44 kcal/mol (±0.03) for **q**_1_ and **q**_2_, respectively. The sampled volume ${\sum }_{site}{e}^{-\beta G(\lambda ,v,\zeta )}\Delta \lambda \Delta v\Delta \zeta$ part of Eq. S2 was evaluated to approximately 0.735 Å^3^ and 0.009 Å^3^ for **q**_1_ and **q**_2_, respectively. This results in a standard binding free energy, $\varDelta {G}_{b}^{0}$, of −15.31 kcal/mol and −12.22 kcal/mol for **q**_1_ and **q**_2_, respectively, showing that **q**_2_ is a weaker binding configuration than **q**_1_. Here, **q**_1_ (our docking prediction) has reproduced the experimental value of ΔG_exp_ = −16.61 kcal/mol^30^.

Figure 2B shows the root-mean-square fluctuation (RMSF) at different regions along λ′ for both the antibody and the Aβ peptide obtained from the path sampling simulations. The complementarity-determining region (CDR) loops of the antibody that form the Aβ binding pocket show to be especially λ′-dependent. Both molecules are very stable in the bound state, where the antibody becomes less stable as the peptide unbinds. Aβ is only stable in the bound state, while in the intermediary and unbound states it fluctuates greatly. Interestingly, the antibody in the intermediary state (λ′ = 10 Å) shows the highest fluctuation, higher than in both the bound (λ′ = 0 Å) and unbound (λ′ = 20 Å) states. Seemingly, the disordered nature of the peptide inflicts increased dynamics upon the antibody, as furthermore illustrated in Fig. 2C. In particular, CDR-L1 (red loop in Fig. 2C) shows increased dynamics, with some structures having Aβ bound near CDR-L1, some structures bound at an intermediary distance (between V_H_ and CDR-L1) and some bound more distantly (near V_H_). The various potential binding configurations of Aβ in this intermediary state greatly influences the antibody, which appears to be destabilized by it. However, these binding configurations are locally stabilized by various interactions forming between Aβ and solanezumab (Movie S1). Thus, due to a large number of weakly stabilizing structural features, a high fluctuation of both Aβ and solanezumab is observed.

To analyze the structures sampled along the binding/unbinding pathway, we constructed a trajectory of representative structures (from the final 100 ns) along λ′ of each bin Δλ from the US simulations. Movie S1 shows how the peptide could potentially bind to (or unbind from) the antibody, where the movie starts from the unbound state, i.e. from a large λ′. In the unbound state (until about λ′ = 18 Å), the Aβ peptide has no contacts with solanezumab. As the peptide approaches the antibody, its contact area with the antibody slowly increases. It starts to form various different non-specific binding configurations that are stabilized within shallow local minima via primarily salt bridges. After the peptide has gotten closer to the CDR loops, some more stable, non-native interactions are starting to be formed. Between λ′ = 9.6 and 8.4 Å, the hydrophobic core of the peptide (Leu17-Ala21) faces inwards towards the binding pocket, interacting with CDR-H1 and CDR-L1, as can also be observed in the PMF as a meta-stable region (Fig. 2A). At λ′ = 7.6 Å, the C-terminal side of the peptide interacts with CDR-H1 where it stays until about λ′ = 6.9 Å, when the C-terminal side moves deeper into the binding site nearby CDR-L3 and CDR-H3. At λ′ = 6.3 Å, a configurational change is observed, as the N-terminal side is now facing towards CDR-L2, with the hydrophobic Val18 and Phe19 facing towards the binding pocket, which is the first step in forming the natively bound contacts. Next, between λ′ = 5.0 and 4.4 Å, Glu22 and Asp23 form fleeting salt bridges to Lys16 and ArgH31 (in CDR-H1), while the C-terminal side is still failing about randomly as it points towards the bulk. At λ′ = 4.2 Å, the salt bridge between Lys16 and AspH100 (in CDR-H3) is formed, anchoring the peptide, to pull it in further. Simultaneously, Phe19 changes its rotamer and enters deep into the pocket, locking it into its place. At λ′ = 3.6 Å, Phe20 also enters deep into the pocket, but it still moves around considerably, with it leaving between λ‘ = 1.8 to 1.5 Å, before it stabilizes and locks into place. As the peptide approaches λ′ = 0 Å, the C-terminal region folds into an α-turn similar to the experimental structure, with the sidechains making various contacts to the antibody. As the peptide is pulled in further into the pocket, deeper than the native state, CDR-L1 somewhat closes up behind it, partially encapsulating the peptide inside the pocket. Here, the C-terminal region faces towards CDR-H2, with the mid-region and C-terminal end of Aβ not significantly different compared to the native state at λ′ = 0 Å. The binding of Aβ also has a considerable impact on the structural ensemble of solanezumab, as can be seen in Fig. 2B, where the RMSF of solanezumab is plotted for different regions along λ′ of the US trajectory. As Aβ binds, solanezumab becomes more stable, as in particular the CDR regions have become more stable in the bound state. This can also be observed in the movie, as the antibody remains unstable until the peptide has sufficiently entered the pocket around λ′ = 4 Å, where it starts to form native contacts with the antibody, stabilizing both molecules.

To investigate the conformational ensemble of the Aβ peptide in isolation, we executed additional McMD simulations of Aβ in solvent, without the antibody. Fig. S7A shows a very wide and shallow landscape without any deep minima, where the reweighted canonical ensemble at room temperature mainly consists of random conformations, which was previously also shown by Baumketner *et al*.^33^. The canonical ensemble at room temperature of Aβ in the presence of solanezumab (i.e. our docking ensemble) projected onto the same two principal components shows a funneled landscape with two major minima (Fig. S7B), where these minima correspond to **r**_1_ and **r**_2_. Thus, in isolation, the peptide monomer forms disordered structures, while upon binding, Aβ is stabilized by forming a hydrophobic core with its central Phe19-Phe20 residues, which is a common structural feature between **r**_1_ and **r**_2_ (panels 1 and 2 in Fig. S3, respectively).

With the lower affinity of the peptide **q**_2_ with respect to **q**_1_ even with its increased stability, it would be interesting to see the stability of the full-length sequence. For that purpose, we constructed two full-length models of both **r**_1_ and **r**_2_. The R-values from the canonical simulations are shown in Figs. S8–S11, with the averages and standard deviations over the final 40 ns of each trajectory shown in Table S7. The data shows that the models derived from **r**_1_ (Figs. S8–S9) have a higher stability than those derived from **r**_2_, (Figs. S10–S11) suggesting once more that **r**_1_ is indeed the most stable one, while **r**_2_ is simply an artifact caused due to the usage of the epitope instead of the full-length sequence.

## Discussion

**Figure 3:**
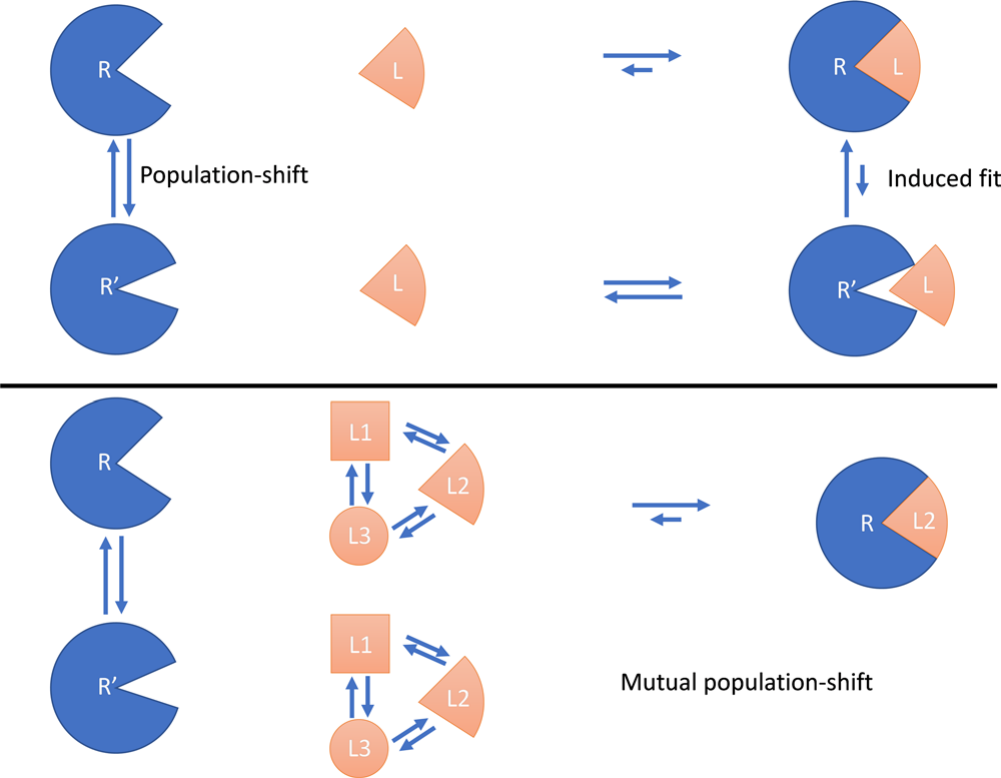
Schematic representation of the existing population-shift and induced-fit models (top panel) and our model, mutual population-shift (bottom panel). For the bottom panel, in the unbound state (left side), populations of various conformations of both the protein (R and R‘) and the ligand (L1, L2 and L3) exist. Both the protein and the ligand require a specific conformation to bind, which upon attaining those, leads to binding (right side) and a population shift of both molecules to R and L2, respectively. The arrows indicate the direction of the conformational/configurational change, where the length of the arrow indicates the relative weight.

The conformational differences between solanezumab in the bound and unbound states observed during our path sampling simulations (Fig. 2C), suggest that Aβ binding to solanezumab is governed by a conformational selection model. Ma *et al*. showed that through analysis of registered protein structures in the PDB that antibodies primarily use the conformational selection model to recognize Aβ antigens^34^. However, due to the flexible nature of the Aβ-peptide, the effects of binding on the peptide itself must also be considered. Aβ, which favors random configurations in solution as shown in Fig. S7A and shown by Baumketner *et al*.^33^, clearly prefers a specific conformation upon binding (Fig. S7B), which would suggests that the opposite, i.e. solanezumab binding to Aβ, is also governed by a conformational selection model. Due to the conformational selection of both molecules, each undergoes a major population-shift from a relatively random conformational ensemble to a single, stable conformation. With these population-shifts occurring roughly simultaneously, an effect that we dub the mutual population-shift is observed, where both molecules impact the dynamics of their reciprocal one (Fig. 3). This mutual population-shift also explains their affinity. The two molecules only have few explicit interactions in the native bound state (i.e., the strong AspH100-Lys16 salt bridge and the relatively weak pi-interactions of Phe19 and Phe20 with the antibody, panel 1 in Fig. S3), but they fit together perfectly, with many minor compounding enthalpic contributions. In addition, the complex formation shields the residues on the interface, reducing the entropic effects of the bulk on both the antibody and the peptide. Combined, they result in both higher stability and affinity as both molecules are stabilized towards a single conformation. Finally, their structure and binding mechanism also explains the specificity of solanezumab for monomeric Aβ. Only Aβ in its monomeric form can sufficiently stabilize solanezumab, as an oligomeric Aβ would not be able to bind sufficiently deep within the binding pocket to induce a conformational shift. Furthermore, the fibril structure of Aβ^35^ forms a β-sheet between the chains and would thus prevent both core Phe-residues of the epitope from binding inside their respective deep pockets, as they face different directions in that form^36^.

To date, the molecular recognition between receptor proteins and their ligand has been widely investigated, and the binding mechanisms can generally be split into the lock-and-key, induced-fit, and conformational shift models as shown in the Introduction section. The recognition becomes more complicated when the size of the protein and/or ligand become(s) larger. For example, a blend of conformational selection with the induced-fit model explains the binding of ubiquitin^37^, which has also been found applicable to the interaction between c-Src kinase and the small chemical drug Imatinib^38^. Another example is the mutually induced conformational change in both calmodulin and its target proteins, which is required to achieve both high affinity and high specificity^39^. On the other hand, in intrinsically-disordered proteins (IDPs), which bind structured partner molecules, the induced-folding, or folding-upon-binding, mechanism is dominant^40,41^, but combined conformational selection with folding-upon-binding was found to direct the redox switch CP12^42^. In IDPs that do not form ordered complex structures, a fuzzy-complex-formation mechanism is also observed, e.g. between Prothymosin α and its partner protein^43^. Our mutual population-shift model can be added to this list of complex models, where two population shift mechanisms are observed as both molecules affect the conformational ensemble of their reciprocal one. Thus, our model complements the known binding mechanisms between large molecules and shows a novel binding mechanism between antibody and antigen.

In conclusion, our multicanonical docking simulations were able to accurately sample the conformational space of solanezumab and Aβ, leading to the natively bound configuration (RMSD = 1.83 Å, R-native = 0.924 with respect to the experimental structure, Table 1), representative binding pathways and, by virtue of additional path sampling simulations, to an accurate estimate of the binding free energy ($\varDelta {G}_{b}^{0}$ = −15.31 kcal/mol, ΔG_exp_ = −16.61 kcal/mol^30^). The structural analysis on the solanezumab and Aβ conformations sampled during the path sampling simulations showed increased dynamics in the unbound and intermediary binding states (λ′ = 20 Å and λ′ = 10 Å, respectively, in Fig. 2B), where a variety of partly bound configurations were sampled at relatively low stability and affinity (Fig. 2). Furthermore, our simulations of Aβ in isolation showed that it behaves like a disordered peptide (Fig. S7A), as shown by its shallow landscape. Taken together, Aβ-solanezumab binding is driven by our novel mutual population-shift model, stabilizing both molecules, which plays a crucial role in their specificity as well as their affinity.

Due to the vast conformational space of the binding pathway, such simulations cannot be performed using conventional MD simulations and require an enhanced sampling method such as McMD. As we have shown the efficacy of our dynamic docking method using increasingly flexible systems starting from small compounds^8,13^, medium-sized compounds^14^ and now peptide-ligands, our future goals will focus on sampling even larger, more complex systems. Similarly, as we focused here on solanezumab, it might also be interesting to investigate whether the mutual population-shift model applies to other therapeutic antibody-antigen targets.

## Methods

### Computational system for the dynamic docking

The computational system for the dynamic docking was built in a similar way as our previous work^13,14^. The Aβ peptide complexed with the antibody was taken from PDB ID 4xxd^36^, whose *auth_asym_id*s (the chain ID in the PDBx/mmCIF notation)^44^ A, B and C corresponding to the V_L_ (light chain), V_H_ (heavy chain) and Aβ were used. The asymmetric unit consists of two biological units, where the Aβ molecule in the second unit (consisting of the chain IDs D, E and F) is missing the C-terminal Gly25 and Ser26 residues. As such, the first biological unit was used. The antibody’s non-F_v_ (with the F_v_ being the variable domain) part of the F_ab_ (antigen binding region), i.e. the C-terminal part of both the V_H_ and V_L_, was removed and replaced with NME-caps. Next, the system was rotated based on its principal axes of inertia, so that the Aβ-binding pocket of the antibody became aligned with the yz-plane. Similar to our previous work^13,14^, we applied cylindrical restraints, and since the antibody was pre-aligned with the yz-plane, the cylinder axis $\mathop{{\rm{\lambda }}}\limits^{\rightharpoonup }$ was set to coincide with the x-axis, as shown in Fig. 1A. The center of the pocket was determined by calculating the COM of the antibody residues nearby (within 10 Å) the Aβ peptide, giving a point deep inside the antibody, which corresponds to λ = 0 Å. Using this pocket center, a cylinder ranging from λ = 2.5 Å (closer to the surface of the pocket) to λ = 30.0 Å (in the bulk region), in combination with a cylinder radius of 4 Å was used during the dynamic docking. Here, the position of Aβ in the experimental structure corresponds to λ = 6.6 Å. Harmonic restraints on the COM of the Aβ peptide perpendicular (beyond the cutoff radius) and parallel (beyond the cutoff range) to the axis $\,\mathop{{\rm{\lambda }}}\limits^{\rightharpoonup }$ were set to 1.0 kcal/mol Å^−^^2^ and 20.0 kcal/mol Å^−^^2^, respectively. To prevent unfolding at high temperatures during the McMD based dynamic docking, we employed position restraints similar to our previous work^13^, where we applied restraints on the heavy atoms of the residues located beyond the reach of Aβ restrained in the cylinder. Here we have restrained only those residues whose sidechain is completely outside the cutoff range, which was defined as 4 Å (cylinder radius) + 9 Å (radius of gyration of Aβ) + 2 Å (buffer region) from the axis $\mathop{{\rm{\lambda }}}\limits^{\rightharpoonup }$. These non-pocket heavy atoms were then position restrained to their initial structure with a force constant of 1.0 kcal/mol Å^−^^2^, and are shown as red in Fig. 1A, while the remaining atoms, particularly the CDR loops, remained flexible.

### McMD simulations of Aβ in isolation

To study the effect of Aβ in isolation and compare the effect of binding to the conformational ensemble, we executed additional McMD simulations of Aβ without solanezumab. The Aβ peptide was placed in a dodecahedron box with a diameter of 60 Å, filled with TIP3P waters and 0.1 M of Na and Cl ions and was prepared and executed in a similar manner as the complex system (producing a total production run of 2.4 µs). Finally, PCA was performed using the distance matrix of the Aβ Cα atoms and Lys16-Nζ, Leu17-Cγ, Phe19-Cζ, Phe20-Cζ, Glu22-Cδ, Asp23-Cγ, Ser26-Oγ, excluding the pairs of ± 3 neighboring residues.

### Generating full-length models of Aβ from r_1_ and r_2_

To assess the stability of **r**_1_ and **r**_2_ considering the full-length sequence, we first generated two full-length initial models of Aβ for both **r**_1_ and **r**_2_ using Modeller^45^, followed by MD simulations to further refine them. We placed the initial models in a dodecahedron box with a diameter of 80 Å with TIP3P waters and 0.1 of Na and Cl ions and performed energy minimization and 100 ps of NVT- and NPT-MD simulations. We then performed a 10 ns NVT MD simulation at 400 K, followed by 1 ns of annealing to 300 K and finally 89 ns at 300 K to relax each model, where a representative structure (nearest-to-average structure over the final 40 ns of the trajectory) was taken for each model. This provided us with four final models; two from **r**_1_ and two from **r**_2_. Then, for each model we performed ten times 100 ns NVT MD simulations at both 300 K and 400 K to estimate the stability similar to our previous work^31^, from which we calculated the R-values (with respect to the representative structure of the corresponding model).

## Supplementary information

Supplementary Information.

## Data Availability

The representative structures and interactive versions of Figs. 1A, 2C, S3,S5,S6 and Movie S1 have been submitted to the Biological Structure Model Archive under BSM-ID BSM00008 (https://bsma.pdbj.org/entry/8).
