# Supplementary material for: Mutual population-shift driven antibody-peptide binding elucidated by molecular dynamics simulations
Source: Sci Rep. 2020 Jan 29;10:1406. doi: 10.1038/s41598-020-58320-z (PMC6989527; doi:10.1038/s41598-020-58320-z)
Supplement: Supplementary file 1 — Supplementary Information. [file 41598_2020_58320_MOESM1_ESM.pdf]

Mutual population-shift driven antibody-peptide binding elucidated by molecular dynamics simulations

Gert-Jan Bekker, **Ikuo Fukuda**, **Junichi Higo**, and **Narutoshi Kamiya**

Table of contents

Section S1	Preparation of the computational system	2
Section S2	McMD simulation for dynamic docking	2
Section S3	Binding/unbinding path generation: picking representative structures from McMD	2
Section S4	Path sampling and affinity calculation by US and WHAM	3
Section S5	Supplementary references	3
Table S1	System sequence information	6
Table S2	US window parameters	7
Table S3	Validation of structures	8
Table S4	Comparison of the R-values and RMSDs of the picked structures between neighboring windows	9
Table S5	PMF and error estimates using different time ranges of the US trajectories starting from $\mathbf{q}_1$	10
Table S6	PMF and error estimates using different time ranges of the US trajectories starting from $\mathbf{q}_2$	11
Table S7	Stability of full-length Aβ models	12
Figure S1	Potential energy probability distribution ($P_{McMD}(E)$) as sampled during the production run	13
Figure S2	FEL of the peptide-solanezumab structures projected onto the first and second principal components	14
Figure S3	Representative structures $\mathbf{r}_k$ predicted by McMD based dynamic docking	15
Figure S4	R-value plots of the canonical MD simulations at 300 K and 400 K of each $\mathbf{r}_k$	16
Figure S5	Refined structures $\mathbf{q}_k$ obtained from the canonical simulations starting from $\mathbf{r}_k$	17
Figure S6	Initial structures that seeded the US simulations and were picked from the McMD ensemble	18
Figure S7	FEL obtained from the conformational ensemble of Aβ in isolation	19
Figure S8	R-value plots of the first full-length model of Aβ produced from $\mathbf{r}_1$	20
Figure S9	R-value plots of the second full-length model of Aβ produced from $\mathbf{r}_1$	21
Figure S10	R-value plots of the first full-length model of Aβ produced from $\mathbf{r}_2$	22
Figure S11	R-value plots of the second full-length model of Aβ produced from $\mathbf{r}_2$	23
Movie S1	https://youtu.be/QYvcKgvdrsQ	

Section S1: Preparation of the computational system

The system was placed into a solvated water box with Na and Cl ions added to match a concentration of 0.1 M, and subsequently subjected to energy minimizations and short NVT- and NPT-MD simulations to equilibrate the system using all-heavy solute position restraints. The final system consists of 27225 atoms in a triclinic box of the dimensions 73.7x69.1x53.1 Å^3. V_H, V_L and Aβ consists of 1624, 1684 and 180 atoms, respectively, with the bulk consisting of 17 Na ions, 20 Cl ions and 7900 water molecules. We performed high temperature simulations corresponding to 700 K employing the restraints described in the main text (cylinder restraints on the COM of Aβ and position restraints on the non-pocket heavy atoms of solanezumab) and used the final structure of this high temperature simulation (Fig. 1A) as the initial structure for our dynamic docking simulations, which are described below. Gromacs 2018[1] was used to execute the simulations, which was extended to execute McMD dynamic docking and path sampling simulations[2]. The AMBER99SB-ildn force field[3], optimized monovalent ion parameters[4] and TIP3P waters[5] were used to parameterize the molecules. LINCS[6] and SETTLE[7] were used to constrain the protein with the Bussi[8] thermostat and the Parrinello-Rahman[9] barostat where applicable. The long-range electrostatics were calculated using the Zero-Dipole summation method[2,10,11] and the cutoff for the electrostatics and van der Waals was set to 12 Å.

Section S2: McMD simulation for dynamic docking

The McMD simulation consist of pre-runs to iteratively estimate the density of states, followed by a production run to sample complex structures. This enables the simulation to perform a random walk within the energy space, leading to enhanced conformational sampling, where in our application of dynamic docking it enables us to efficiently sample the binding/unbinding pathway. Since the McMD algorithm has been thoroughly described in many of our previous works[2,12–20], we will instead focus on our protocol. To generate a structure where Aβ is located far from the antibody, the system was first simulated using a high temperature MD simulation at 700 K with position restraints applied to the non-pocket region of solanezumab. Afterwards, 24 parallel simulations were initialized from this structure with different randomized velocities and subjected to a 100 ps NVT simulation at 300 K. Then, a 1 ns MD simulation corresponding to 700 K was performed and the potential energies were sampled, from which the initial bias for McMD was estimated. Subsequent iterations to update the bias were executed until a flat potential energy distribution was obtained. During the production run, the potential energy (E) at each step was stored in addition to the full structures at every 5 ps. The structures sampled during the McMD simulations were reweighted using the canonical probability function at a given temperature T, $P_c(E, T)$. For the analysis of the McMD simulations, the free energy landscape (FEL) at 300 K along the first and second principal components (PC1 and PC2, respectively) from the Principal Component Analysis (PCA) was calculated. The distance matrix of the Cα atoms of the antibody versus the Cα atoms of Aβ and the intramolecular distance matrix of the Cα atoms of Aβ, were used to perform the PCA. From the FEL, we extracted the bins with a free energy value less than 25 kcal/mol, after which we used K-means clustering to identify k=10 clusters. A representative structure close to the center of each cluster was picked (taking into account each structure's weight $P_c(E, T)$), producing 10 representative meta-stable configurations $\mathbf{r}_k$. To refine and validate our obtained meta-stable structures, we performed 100 ns NVT-MD simulations at 300 K and 400 K, starting from each $\mathbf{r}_k$, without position and cylinder restraints, where the translation and rotation of the COM of solanezumab were restrained like our previous works[2,20].

Recently, we showed that the relative stability of single chain antibodies can be estimated by calculating the average Q-value[21] (i.e. the fraction of native contacts) along MD trajectories[22]. We also introduced a new quantity, R, which calculates the fraction of contacts between two molecules with respect to a reference structure, where a value of 1.0 means that the complex forms the same interactions between these two molecules as in the reference structure[20]. The Q-value is defined as $Q = \frac{1}{N}\sum_{(i,j)} \frac{1}{1+\exp(\beta(r_{ij}(X)-\Lambda r_{ij}^0))}$, where N is the number of native contact pairs with a distance less than 4.5 Å, $r_{ij}(X)$ the distance of the pair (i, j) in configuration X, r_{ij}^0 the distance in the native configuration, β a smoothing parameter and Λ a parameter to account for the fluctuations formed by the contact, set to 5.0 Å^{-1} and 1.8 Å, respectively[21]. The R-value distinguishes from the Q-value in the definition of i and j and the used structure. Whereas for the Q-value i and j correspond to atoms from the whole structure, for the R-value i corresponds to atoms taken from the protein and j to atoms taken from the ligand. Finally, any reference structure's distance r_{ij}^{ref} can be used instead of the native structure's distance r_{ij}^0 for the R-value, where for the notation of R(native)-value the experimental "native" structure is used as a reference structure, but still differs from the Q-value by only including the atom pairs between the protein and the ligand. We then calculated the R-value of each trajectory of the canonical simulations with respect to their initial structure $\mathbf{r}_k$ to evaluate the stability. The R-value thus measures how well the intermolecular interactions are maintained during canonical MD simulations at both room temperature (i.e. 300 K) and high temperature (i.e. 400 K), providing an assessment regarding the stability of each structure $\mathbf{r}_k$.

Section S3: Binding/unbinding path generation: picking representative structures from McMD

We previously described a structure picking method that can pick structures from an McMD trajectory to generate a smooth path in our recent docking between β-secretase and its inhibitor[20]. For clarity we will shortly review it here. First, for the most stable structure based on the FEL (i.e. $\mathbf{r}_1$), we generated a representative equilibrated structure based on the final 40 ns of the canonical trajectory at 300 K by taking the nearest-to-average structure to produce $\mathbf{q}_1$, which will serve as the starting point for our affinity calculations. Next, we defined λ′ as λ′ = λ – 6.96 Å, where 6.96 Å corresponds to the λ value of the structure $\mathbf{q}_1$, making it easier to define parameters relative to this structure. Next, we defined 16 windows from which to pick representative structures from, where we picked 3 unique structures per window to increase the statistics of the US simulations. For the picking process, each window is defined by the parameters λ'_l, λ'_c, λ'_u along $\vec{\lambda}$ as described in

Table S2. For each window, we first obtain a set of eligible structures by filtering the entire McMD ensemble based on the λ' range λ'_l - λ'_u. Next, we filter them based on the structural similarity with respect to the picked structures from the preceding window via a nearest neighbor like approach, obtaining a filtered ensemble. Finally, using this subset, we performed K-means clustering (with k=3) where we take a representative structure from each cluster. This leads to an optimally structurally connected ensemble of structures along λ' from the predicted bound configuration to the unbound state, given the McMD ensemble and starting configuration. We performed this procedure for both $\mathbf{q}_1$ (the most stable structure predicted by our dynamic docking, followed by equilibration MD) and $\mathbf{q}_2$ (the structure suggested by our stability testing as being highly stable, although it is an unrealistic one considering the full-length sequence), where we performed the same picking procedure starting from $\mathbf{q}_2$ instead of $\mathbf{q}_1$ to generate a pathway to connect $\mathbf{q}_2$ to the unbound state along λ'. Table S4 lists the similarity between the structures from neighboring windows for $\mathbf{q}_1$.

Section S4: Path sampling and affinity calculation by US and WHAM

The picked structures were then used for the path sampling simulations using Umbrella Sampling (US). For the reaction coordinate, the axis $\vec{\lambda}$, which corresponds to the x-axis of the computational system, was used. The picked structures were then restrained so that the structure maintains its λ-coordinate (λ'_c) of the corresponding window (Table S2), while perpendicular to this axis no restraints were applied, allowing the peptide to sample freely, except along the reaction coordinate. Before the production run of US, first a 100 ps NVT-MD simulation with position restraints on the heavy solute atoms was executed, with random velocities set at 400 K. Next, a 1.8 ns NVT simulation at 400 K was executed without the position restraints, followed by 100 ps of annealing to 300 K and finally 100 ps of NVT at 300 K. The initial equilibration simulations executed at 400 K enables the during the McMD simulation's restrained system to anneal into a relaxed configuration significantly faster than if they were executed entirely at 300 K, as we showed previously[20]. The production US simulations to measure the physical properties were executed at room temperature, i.e. at 300 K, lasting 200 ns for each parallel simulation. During the 2 ns equilibration and the 200 ns US simulations, the COM of Aβ was restrained along $\vec{\lambda}$ with an umbrella potential to the center of the corresponding window (λ'_c) using the force constant as described in Table S2, while the peptide was not restrained perpendicular to the axis $\vec{\lambda}$. The translation and rotation of the antibody's COM were restrained as described above, without any position restraints. Finally, the PMF along λ' was calculated using the Weighted Histogram Analysis Method (WHAM)[23,24], which was performed using a bin size Δλ of 0.05 Å, a tolerance of 1×10^{-8} and with 1000 bootstraps to estimate the error, where the binding free energy ΔG was then calculated as follows:

$$\Delta G = -\big(G(\lambda_\infty) - G(\lambda_0)\big) \qquad \text{[S1]}$$

where $G(\lambda_\infty)$ corresponds to the estimate in the unbound state and $G(\lambda_0)$ in the bound state. The wide sampling of the US simulations enables us to calculate the sampled volume directly from these simulations to apply the correction term $e^{-\beta G(\lambda,v,\zeta)}$ from the probability distribution of the ensemble sampled during the US simulations, weighted by PMF obtained from WHAM, i.e., the distribution along (v,ζ) was weighted by $G(\lambda)$. Here, v and ζ correspond to the axes perpendicular to $\vec{\lambda}$ and the 3D grid consists of cubic cells of size Δλ = 0.05 Å. Finally, the standard binding free energy ΔG_b^0 was estimated using the following equation:

$$\Delta G_b^0 = \Delta G - k_B T \times \ln\Big(\frac{1}{V_0}\sum_{site} e^{-\beta G(\lambda,v,\zeta)}\Delta\lambda\Delta v\Delta\zeta\Big) \qquad \text{[S2]}$$

where ΔG corresponds to the binding free energy calculated in Eq. S1, V_0 to the standard concentration at 1 mol/L (1661 Å^3), $e^{-\beta G(\lambda,v,\zeta)}$ to the probability of sampling inside the 3D grid cell (λ, v, ζ) and $\Delta\lambda\Delta v\Delta\zeta$ to the volume of the grid cell.

Section S5: Supplementary references

1. Kutzner, C. *et al.* More bang for your buck: Improved use of GPU nodes for GROMACS 2018. *J. Comput. Chem.* **40**, 2418–2431 (2019).

2. Bekker, G.-J. *et al.* Accurate Prediction of Complex Structure and Affinity for a Flexible Protein Receptor and Its Inhibitor. *J. Chem. Theory Comput.* **13**, 2389–2399 (2017).

3. Lindorff-Larsen, K. *et al.* Improved Side-Chain Torsion Potentials for the Amber ff99SB Protein Force Field. *Proteins* **78**, 1950–1958 (2010).

4. Joung, I. S. & Cheatham, T. E. Determination of alkali and halide monovalent ion parameters for use in explicitly solvated biomolecular simulations. *J. Phys. Chem. B* **112**, 9020–9041 (2008).

5. Jorgensen, W. L., Chandrasekhar, J., Madura, J. D., Impey, R. W. & Klein, M. L. Comparison of Simple Potential Functions for Simulating Liquid Water. *J. Chem. Phys.* **79**, 926–935 (1983).

6. Hess, B. P-LINCS: A Parallel Linear Constraint Solver for Molecular Simulation. *J. Chem. Theory Comput.* **4**, 116–122 (2008).

7. Miyamoto, S. & Kollman, P. A. Settle - An Analytical Version of the Shake and Rattle Algorithm for Rigid

Water Models. *J. Comput. Chem.* **13**, 952–962 (1992).

8. Bussi, G., Donadio, D. & Parrinello, M. Canonical Sampling Through Velocity Rescaling. *J. Chem. Phys.* **126**, 14101 (2007).

9. Parrinello, M. & Rahman, A. Polymorphic Transitions in Single-Crystals - A New Molecular-Dynamics Method. *J. Appl. Phys.* **52**, 7182–7190 (1981).

10. Fukuda, I., Yonezawa, Y. & Nakamura, H. Molecular dynamics scheme for precise estimation of electrostatic interaction via zero-dipole summation principle. *J. Chem. Phys.* **134**, 164107 (2011).

11. Kamiya, N., Fukuda, I. & Nakamura, H. Application of zero-dipole summation method to molecular dynamics simulations of a membrane protein system. *Chem. Phys. Lett.* **568**–**569**, 26–32 (2013).

12. Nakajima, N., Nakamura, H. & Kidera, A. Multicanonical Ensemble Generated by Molecular Dynamics Simulation for Enhanced Conformational Sampling of Peptides. *J. Phys. Chem. B* **101**, 817–824 (1997).

13. Nakajima, N. A selectively enhanced multicanonical molecular dynamics method for conformational sampling of peptides in realistic water molecules. *Chem. Phys. Lett.* **288**, 319–326 (1998).

14. Nakajima, N., Higo, J., Kidera, A. & Nakamura, H. Free Energy Landscapes of Peptides by Enhanced Conformational Sampling. *J. Mol. Biol.* **296**, 197–216 (2000).

15. Kamiya, N., Yonezawa, Y., Nakamura, H. & Higo, J. Protein-Inhibitor Flexible Docking by a Multicanonical Sampling: Native Complex Structure with the Lowest Free Energy and a Free-Energy Barrier Distinguishing the Native Complex from the Others. *Proteins* **70**, 41–53 (2008).

16. Higo, J., Nishimura, Y. & Nakamura, H. A Free-Energy Landscape for Coupled Folding and Binding of an Intrinsically Disordered Protein in Explicit Solvent from Detailed All-Atom Computations. *J. Am. Chem. Soc.* **133**, 10448–10458 (2011).

17. Ikebe, J. *et al.* Theory for Trivial Trajectory Parallelization of Multicanonical Molecular Dynamics and Application to a Polypeptide in Water. *J. Comput. Chem.* **32**, 1286–1297 (2011).

18. Higo, J., Ikebe, J., Kamiya, N. & Nakamura, H. Enhanced and Effective Conformational Sampling of Protein Molecular Systems for Their Free Energy Landscapes. *Biophys. Rev.* **4**, 27–44 (2012).

19. Nishigami, H., Kamiya, N. & Nakamura, H. Revisiting Antibody Modeling Assessment for CDR-H3 Loop. *Protein Eng. Des. Sel.* **29**, 477–484 (2016).

20. Bekker, G.-J., Araki, M., Oshima, K., Okuno, Y. & Kamiya, N. Dynamic Docking of a Medium-Sized Molecule to Its Receptor by Multicanonical MD Simulations. *J. Phys. Chem. B* **123**, 2479–2490 (2019).

21. Best, R. B., Hummer, G. & Eaton, W. A. Native Contacts Determine Protein Folding Mechanisms in Atomistic Simulations. *Proc. Natl. Acad. Sci. USA* **110**, 17874–17879 (2013).

22. Bekker, G. J., Ma, B. & Kamiya, N. Thermal stability of single-domain antibodies estimated by molecular dynamics simulations. *Protein Sci.* **28**, 429–438 (2019).

23. Kumar, S., Bouzida, D., Swendsen, R. H., Kollman, P. A. & Rosenberg, J. M. The Weighted Histogram Analysis Method for Free-Energy Calculations on Biomolecules. I. The Method. *J. Comput. Chem.* **13**, 1011–1021 (1992).

24. Grossfield, A. WHAM: An Implementation of the Weighted Histogram Analysis Method.

25. Adolf-Bryfogle, J., Xu, Q., North, B., Lehmann, A. & Dunbrack, R. L. PyIgClassify: a database of antibody CDR structural classifications. *Nucleic Acids Res.* **43**, D432–D438 (2015).

26. Bekker, G.-J., Nakamura, H. & Kinjo, A. R. Molmil: a molecular viewer for the PDB and beyond. *J. Cheminform.* **8**, 42 (2016).

27. Kinjo, A. R. *et al.* Protein Data Bank Japan (PDBj): updated user interfaces, resource description framework, analysis tools for large structures. *Nucleic Acids Res.* **45**, D282–D288 (2017).

28. Kinjo, A. R. *et al.* New tools and functions in data-out activities at Protein Data Bank Japan (PDBj). *Protein Sci.* **27**, 95–102 (2018).

Table S1: System sequence information[a]

Name	Sequence	Range
CDR-L1	RSSQSLIYSDGNAYLH	ArgL24-HisL39
CDR-L2	YKVSNRFS	TyrL54-SerL61
CDR-L3	SQSTHVPWT	SerL94-ThrL102
CDR-H1	AASGFTFSRYSMS	AlaH23-SerH35
CDR-H2	QINSVGSSTY	GlnH50-TyrH59
CDR-H3	ASGDY	AlaH97-TyrH101
Aβ	KLVFFAEDVGS	Lys16-Ser26

[a] Sequence information of the CDR loops of solanezumab and the Aβ peptide. The sequences and the corresponding ranges of the loops were obtained from Dunbrack's PyIgClassify database[25]. The residue numbers part of V_H start with H, while those part of V_L start with L, otherwise they are part of the Aβ peptide.

Table S2: US window parameters [a]

**Window ID**	**Lower λ'_l (Å)**	**Center λ'_c (Å)**	**Upper λ'_u (Å)**	**Force constant (kcal/mol/Å)**
1	-2.50	-2.00	-1.50	10.0
2	-1.50	-1.00	-0.50	10.0
3	-0.50	0.00	0.50	10.0
4	0.50	1.00	1.50	10.0
5	1.50	2.00	2.50	8.5
6	2.50	3.00	3.50	5.5
7	3.50	4.00	5.00	4.0
8	5.00	6.00	7.00	3.5
9	7.00	8.00	9.00	3.5
10	9.00	10.00	11.00	3.5
11	11.00	12.00	13.00	3.5
12	13.00	14.00	15.00	3.5
13	15.00	16.00	17.00	3.5
14	17.00	18.00	19.00	3.5
15	19.00	20.00	21.00	3.5
16	21.00	22.00	23.00	3.5

[a] Window parameters used during the path generation and US simulations. λ'_l, λ'_u and λ'_c are the lower and upper ranges and the center of each window, respectively. Window ID 3 corresponds to the initial window, where the reference structure (cyan structures in Fig. S5) corresponds to either $\mathbf{q}_1$ or $\mathbf{q}_2$. The force constant as used by the US simulations are shown in the last column.

Table S3. Validation of structures $\mathbf{r}_k$

k	R-value [a] 300 K	R-value [a] 400 K
1	0.898 (0.012)	0.880 (0.030)
2	0.996 (0.008)	0.959 (0.017)
3	0.973 (0.019)	0.910 (0.042)
4	0.978 (0.015)	0.897 (0.025)
5	0.984 (0.014)	0.862 (0.025)
6	0.921 (0.026)	0.771 (0.092)
7	0.869 (0.018)	0.861 (0.038)
8	0.960 (0.023)	0.794 (0.050)
9	0.939 (0.022)	0.477 (0.041)
10	0.928 (0.025)	0.908 (0.035)

[a] Average R-values (final 40 ns) along the 300 K and 400 K trajectories of each binding configuration starting from $\mathbf{r}_k$ with their respective standard deviation in parenthesis.

Table S4. Comparison of the R-values and RMSDs of the picked structures between neighboring windows [a]

Window ID	Previous window's structure -> current window's structure R-value (RMSD in Å)								
	**1->1**	**1->2**	**1->3**	**2->1**	**2->2**	**2->3**	**3->1**	**3->2**	**3->3**
**1**	0.847 (5.377)	0.875 (5.155)	0.883 (5.455)	0.879 (5.274)	0.897 (5.600)	0.920 (4.684)	0.918 (5.316)	0.917 (5.646)	0.956 (4.745)
**2**	0.948 (2.233)	0.980 (2.806)	0.969 (2.660)	0.987 (3.018)	0.991 (3.267)	0.954 (2.950)	0.935 (3.796)	0.992 (1.862)	0.916 (1.906)
**3**	1.000 (0.000)	0.957 (3.051)	0.927 (3.572)	X	X	X	X	X	X
**4**	0.977 (2.617)	0.914 (3.838)	0.912 (3.899)	0.999 (2.001)	0.944 (1.865)	0.985 (2.676)	0.933 (3.111)	0.913 (3.066)	0.913 (2.494)
**5**	0.851 (4.028)	0.858 (4.246)	0.864 (4.651)	0.895 (3.698)	0.904 (3.144)	0.869 (5.149)	0.858 (4.389)	0.859 (4.120)	0.840 (5.259)
**6**	0.900 (3.243)	0.756 (3.933)	0.943 (3.653)	0.851 (4.259)	0.672 (4.194)	0.854 (5.008)	0.882 (5.805)	0.860 (4.794)	0.868 (5.338)
**7**	0.372 (5.783)	0.609 (4.758)	0.673 (5.338)	0.385 (7.002)	0.510 (6.718)	0.758 (5.096)	0.354 (5.418)	0.516 (5.101)	0.689 (4.931)
**8**	0.346 (5.382)	0.313 (4.878)	0.779 (3.658)	0.261 (6.138)	0.178 (6.023)	0.400 (5.777)	0.171 (5.552)	0.257 (4.531)	0.436 (5.322)
**9**	0.027 (8.077)	0.136 (7.008)	0.242 (6.815)	0.096 (7.159)	0.258 (6.060)	0.180 (6.496)	0.439 (5.406)	0.350 (6.025)	0.271 (5.785)
**10**	0.107 (6.950)	0.131 (7.730)	X	0.038 (6.323)	0.129 (7.154)	X	0.266 (4.683)	0.404 (5.077)	X
**11**	0.667 (5.442)	0.670 (3.602)	0.996 (2.473)	0.743 (6.381)	0.569 (4.833)	0.882 (4.050)	X	X	X
**12**	0.635 (2.968)	0.993 (2.872)	0.770 (3.074)	0.398 (5.063)	0.911 (4.770)	0.462 (4.164)	0.320 (5.208)	0.495 (5.195)	0.425 (4.580)
**13**	0.205 (5.403)	X	X	0.000 (5.398)	X	X	0.097 (6.210)	X	X
**14**	0.019 (5.177)	0.000 (5.917)	X	X	X	X	X	X	X
**15**	0.000 (7.777)	X	X	0.000 (2.383)	X	X	X	X	X
**16**	0.000 (5.858)	0.000 (5.595)	0.000 (2.546)	X	X	X	X	X	X

[a] Compares the structures from the preceding window (before the arrow) with those from the given window (after the arrow), which are used as the input structures for the US. For unavailable comparisons, "X" is shown instead. For the initial window (i.e. window 3), there is only one reference structure, i.e. $\mathbf{q}_1$ and the comparison of the three listed structure is made with respect to that one, which is why the first structure has an R-value of 1.0 and an RMSD of 0.0 Å. For the unlisted structures (due to a too narrow ensemble) in window 10 (1), 13 (2), 14 (1) and 15 (2), the first structure was replicated for the other seeds from the same window (with a different random seed for the initial velocities).

Table S5. PMF and error estimates using different time ranges of the US trajectories starting from q_1. [a]

Start (ns)	End (ns)	ΔG	σ	ε	ΔG_b^0
0	10	22.35	0.08	0.10	-17.84
0	20	23.58	0.03	0.07	-19.04
0	30	22.72	0.04	0.06	-18.26
0	40	21.65	0.05	0.05	-17.19
0	50	21.47	0.03	0.05	-17.02
0	60	21.57	0.03	0.04	-17.14
0	70	21.57	0.02	0.04	-17.19
0	80	21.37	0.02	0.04	-16.95
0	90	21.40	0.02	0.03	-17.01
0	100	21.04	0.02	0.03	-16.65
0	110	20.90	0.02	0.03	-16.51
0	120	21.03	0.02	0.03	-16.63
0	130	21.02	0.02	0.03	-16.60
0	140	20.72	0.02	0.03	-16.31
0	150	20.60	0.02	0.03	-16.21
0	160	20.64	0.02	0.03	-16.28
0	170	20.53	0.02	0.03	-16.13
0	180	20.55	0.02	0.02	-16.12
0	190	20.46	0.02	0.02	-15.99
0	200	20.38	0.02	0.02	-15.86
10	200	20.24	0.02	0.02	-15.71
20	200	19.98	0.02	0.02	-15.45
30	200	19.90	0.01	0.03	-15.37
40	200	20.00	0.01	0.03	-15.47
50	200	19.98	0.02	0.03	-15.44
60	200	19.84	0.02	0.03	-15.29
70	200	19.77	0.02	0.03	-15.19
80	200	19.72	0.02	0.03	-15.15
90	200	19.67	0.02	0.03	-15.08
**100**	**200**	**19.92**	**0.02**	**0.03**	**-15.31**
110	200	19.96	0.02	0.04	-15.34
120	200	19.67	0.02	0.04	-15.03
130	200	19.51	0.02	0.04	-14.85
140	200	19.83	0.02	0.04	-15.14
150	200	20.02	0.02	0.05	-15.26
160	200	19.80	0.02	0.06	-14.95
170	200	20.23	0.02	0.06	-15.35
180	200	19.83	0.03	0.08	-14.96
190	200	20.48	0.05	0.11	-15.64

[a] All WHAM calculations were executed using the same parameters as the calculations used in the main text (i.e. a Δλ of 0.05 Å, a tolerance of 1×10^{-8} and with 1000 bootstraps). ΔG is the average PMF over the final 50 bins (2.5 Å), with σ its standard deviation. ε corresponds to the average error (via bootstrapping) taken over the same range. Finally, ΔG_b^0 is the standard binding free energy calculated using ΔG and the corresponding sample COMs during the start and end range of the simulation following Eq. S2.

Table S6. PMF and error estimates using different time ranges of the US trajectories starting from q_2. [a]

**Start (ns)**	**End (ns)**	**ΔG**	**σ**	**ε**	**ΔG_b^0**
0	10	18.58	0.04	0.11	-10.74
0	20	18.02	0.03	0.08	-10.09
0	30	17.82	0.03	0.06	-10.09
0	40	17.95	0.03	0.05	-10.37
0	50	18.20	0.04	0.05	-10.72
0	60	18.09	0.03	0.05	-10.68
0	70	18.38	0.02	0.04	-11.03
0	80	18.59	0.02	0.04	-11.28
0	90	18.75	0.02	0.03	-11.41
0	100	18.73	0.02	0.03	-11.36
0	110	18.55	0.02	0.03	-11.18
0	120	18.27	0.02	0.03	-10.93
0	130	18.28	0.02	0.03	-10.96
0	140	18.19	0.01	0.03	-10.90
0	150	18.26	0.01	0.03	-11.01
0	160	18.38	0.02	0.03	-11.11
0	170	18.61	0.02	0.03	-11.34
0	180	18.71	0.02	0.03	-11.42
0	190	18.68	0.02	0.02	-11.39
0	200	18.87	0.02	0.02	-11.57
10	200	18.86	0.02	0.02	-11.60
20	200	18.98	0.02	0.03	-11.76
30	200	19.19	0.02	0.03	-11.97
40	200	19.36	0.01	0.03	-12.14
50	200	19.46	0.01	0.03	-12.23
60	200	19.51	0.01	0.03	-12.27
70	200	19.48	0.02	0.03	-12.22
80	200	19.40	0.02	0.03	-12.12
90	200	19.41	0.02	0.03	-12.15
**100**	**200**	**19.44**	**0.02**	**0.03**	**-12.22**
110	200	19.71	0.02	0.04	-12.50
120	200	20.12	0.02	0.04	-12.89
130	200	20.21	0.03	0.04	-12.97
140	200	20.67	0.03	0.04	-13.36
150	200	20.81	0.04	0.05	-13.40
160	200	20.97	0.03	0.05	-13.56
170	200	20.65	0.03	0.06	-13.23
180	200	20.52	0.03	0.08	-13.19
190	200	21.75	0.04	0.11	-14.35

[a] All WHAM calculations were executed using the same parameters as the calculations used in the main text (i.e. a Δλ of 0.05 Å, a tolerance of 1×10^{-8} and with 1000 bootstraps). ΔG is the average PMF over the final 50 bins (2.5 Å), with σ its standard deviation. ε corresponds to the average error (via bootstrapping) taken over the same range. Finally, ΔG_b^0 is the standard binding free energy calculated using ΔG and the corresponding sample COMs during the start and end range of the simulation following Eq. S2.

Table S7. Stability of full-length Aβ models.[a]

Model	Trajectory	R-value at 300 K		R-value at 400 K	
		**μ**	**σ**	**μ**	**σ**
1st $\mathbf{r}_1$	1	0.936	-0.017	0.657	-0.024
1st $\mathbf{r}_1$	2	0.973	-0.010	0.568	-0.036
1st $\mathbf{r}_1$	3	0.960	-0.009	0.672	-0.036
1st $\mathbf{r}_1$	4	0.988	-0.007	0.396	-0.022
1st $\mathbf{r}_1$	5	0.964	-0.013	0.480	-0.045
1st $\mathbf{r}_1$	6	0.973	-0.013	0.576	-0.054
1st $\mathbf{r}_1$	7	0.961	-0.008	0.587	-0.045
1st $\mathbf{r}_1$	8	0.934	-0.010	0.462	-0.063
1st $\mathbf{r}_1$	9	0.968	-0.011	0.562	-0.056
1st $\mathbf{r}_1$	10	0.959	-0.025	0.562	-0.036
2nd $\mathbf{r}_1$	1	0.927	-0.011	0.617	-0.014
2nd $\mathbf{r}_1$	2	0.982	-0.016	0.782	-0.037
2nd $\mathbf{r}_1$	3	0.883	-0.025	0.825	-0.037
2nd $\mathbf{r}_1$	4	0.898	-0.018	0.737	-0.020
2nd $\mathbf{r}_1$	5	0.943	-0.012	0.729	-0.017
2nd $\mathbf{r}_1$	6	0.978	-0.012	0.898	-0.037
2nd $\mathbf{r}_1$	7	0.968	-0.016	0.874	-0.023
2nd $\mathbf{r}_1$	8	0.913	-0.011	0.713	-0.038
2nd $\mathbf{r}_1$	9	0.927	-0.030	0.825	-0.025
2nd $\mathbf{r}_1$	10	0.941	-0.019	0.923	-0.030
1st $\mathbf{r}_2$	1	0.817	-0.013	0.694	-0.026
1st $\mathbf{r}_2$	2	0.944	-0.016	0.639	-0.027
1st $\mathbf{r}_2$	3	0.927	-0.023	0.625	-0.033
1st $\mathbf{r}_2$	4	0.871	-0.016	0.651	-0.018
1st $\mathbf{r}_2$	5	0.851	-0.022	0.535	-0.034
1st $\mathbf{r}_2$	6	0.874	-0.034	0.665	-0.025
1st $\mathbf{r}_2$	7	0.872	-0.026	0.686	-0.021
1st $\mathbf{r}_2$	8	0.940	-0.017	0.641	-0.024
1st $\mathbf{r}_2$	9	0.947	-0.015	0.709	-0.033
1st $\mathbf{r}_2$	10	0.904	-0.033	0.448	-0.049
2nd $\mathbf{r}_2$	1	0.829	-0.029	0.270	-0.051
2nd $\mathbf{r}_2$	2	0.644	-0.034	0.396	-0.027
2nd $\mathbf{r}_2$	3	0.523	-0.016	0.544	-0.040
2nd $\mathbf{r}_2$	4	0.783	-0.022	0.325	-0.038
2nd $\mathbf{r}_2$	5	0.812	-0.043	0.421	-0.064
2nd $\mathbf{r}_2$	6	0.669	-0.029	0.441	-0.035
2nd $\mathbf{r}_2$	7	0.727	-0.024	0.380	-0.024
2nd $\mathbf{r}_2$	8	0.701	-0.026	0.415	-0.027
2nd $\mathbf{r}_2$	9	0.830	-0.035	0.469	-0.022
2nd $\mathbf{r}_2$	10	0.770	-0.033	0.444	-0.029

[a] Average R-values, μ, (final 40 ns) along the 300 K and 400 K trajectories of each parallel trajectory for each model starting from the equilibrated structures with their respective standard deviation, σ.

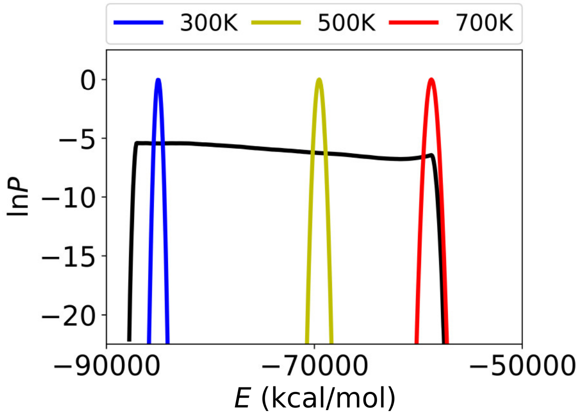

Figure S1. Potential energy probability distribution ($P_{McMD}(E)$) as sampled during the production run. Also shown are the reweighing canonical distributions ($P_c(E, T)$) at 300 K, 500 K and 700 K in blue, yellow and red, respectively.

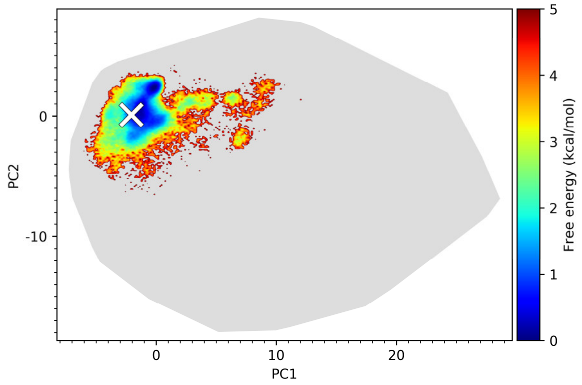

Figure S2. FEL of the peptide-solanezumab structures projected onto the first and second principal components, PC1 and PC2, respectively, where PC1 captures 34.5% and PC2 16.4% of the variation. The sampled region with a free energy higher than 5 kcal/mol corresponds to the unstable region and is represented as a grey convex.

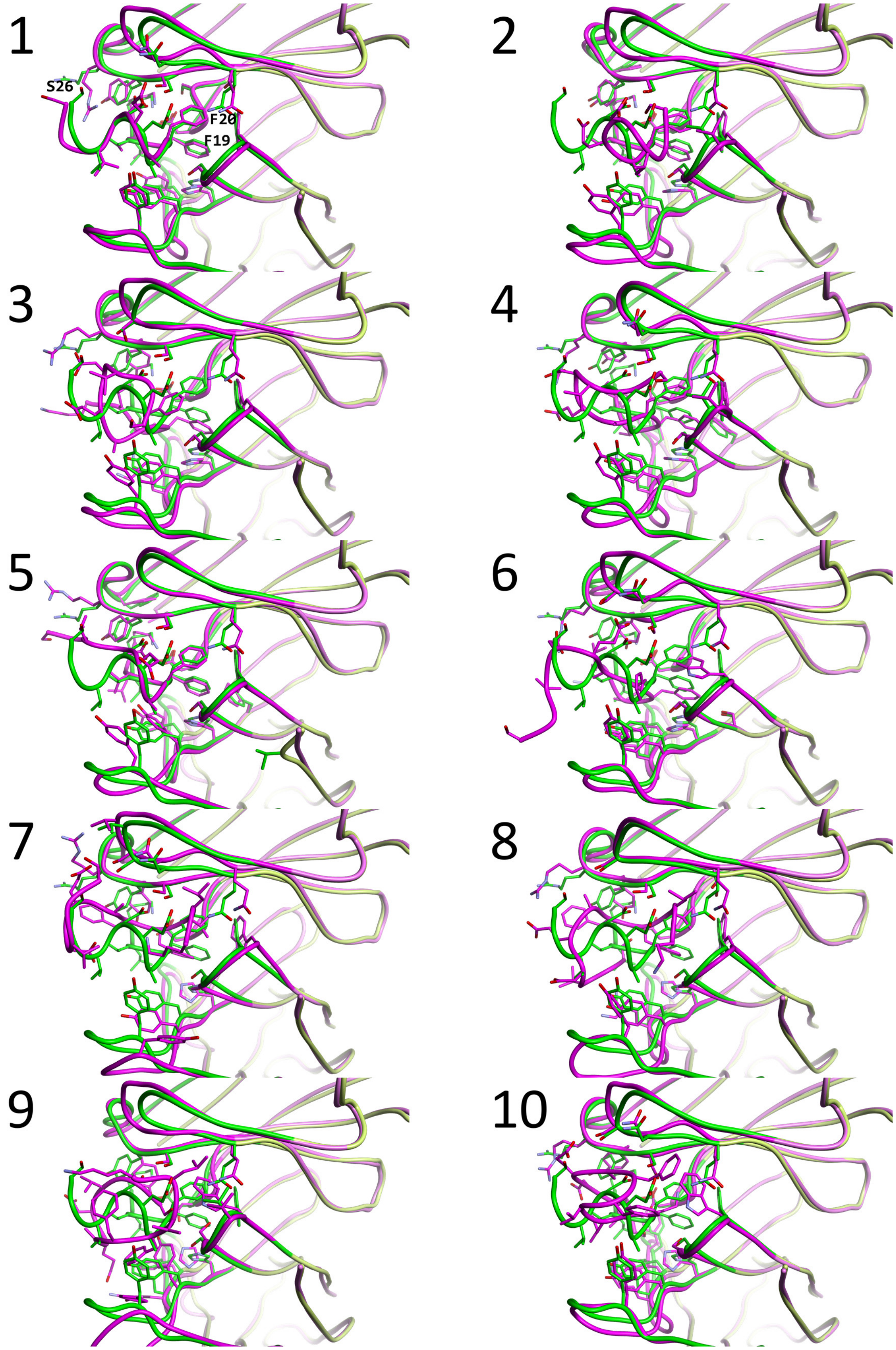

Figure S3. Representative structures $\mathbf{r}_k$ predicted by McMD based dynamic docking. Shown are the structures k=1-10 in magenta and the experimental structure in green. Also shown are the side-chains of solanezumab interacting with Aβ. Finally, for k=1, Phe19, Phe20 and Ser26 are indicated. The images were drawn using Molmil[26], a WebGL based molecular viewer developed by Protein Data Bank Japan[27,28].

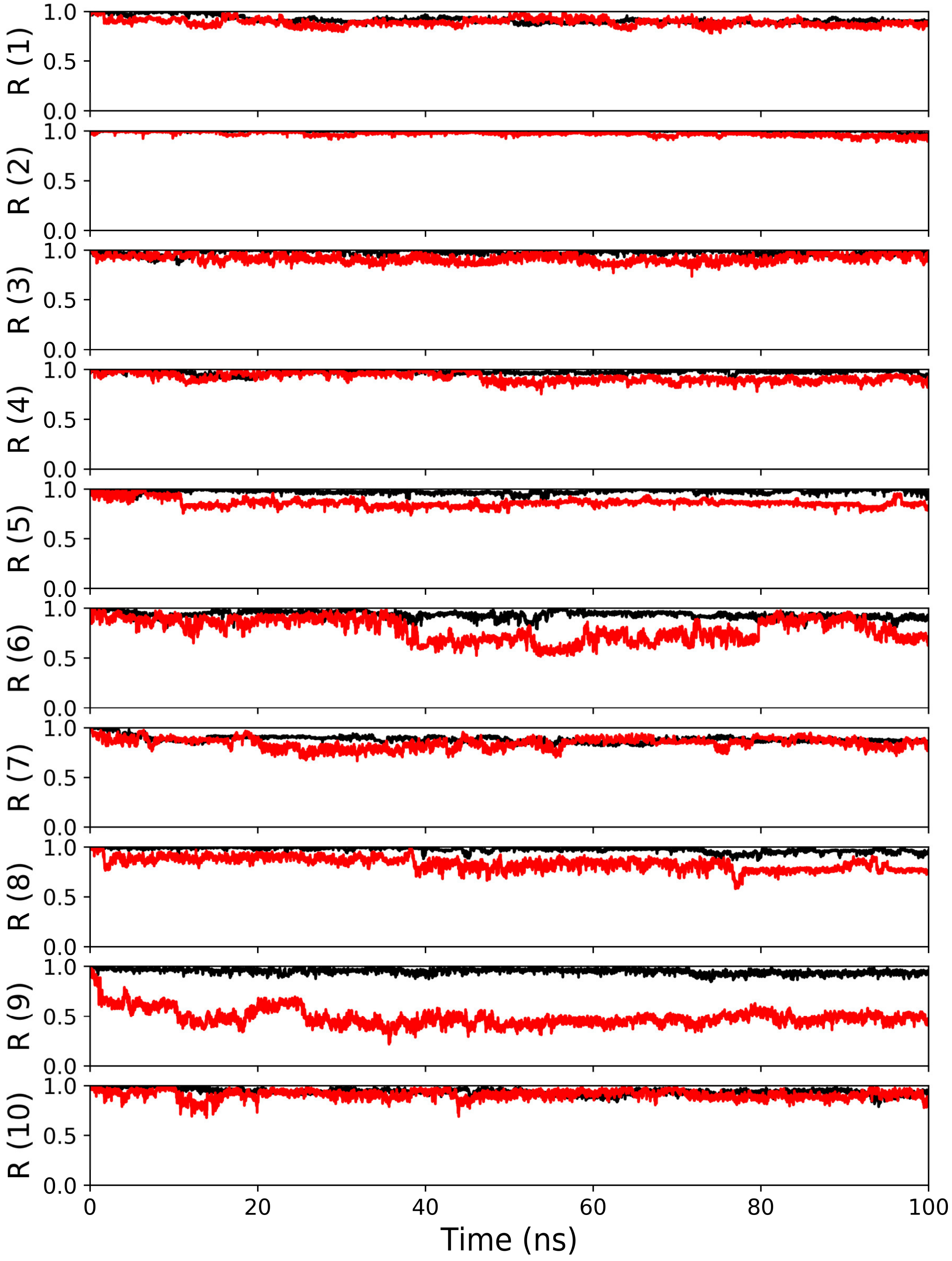

Figure S4. R-value plots of the canonical MD simulations at 300 K and 400 K of each $\mathbf{r}_k$ (k=1-10) in black and red, respectively.

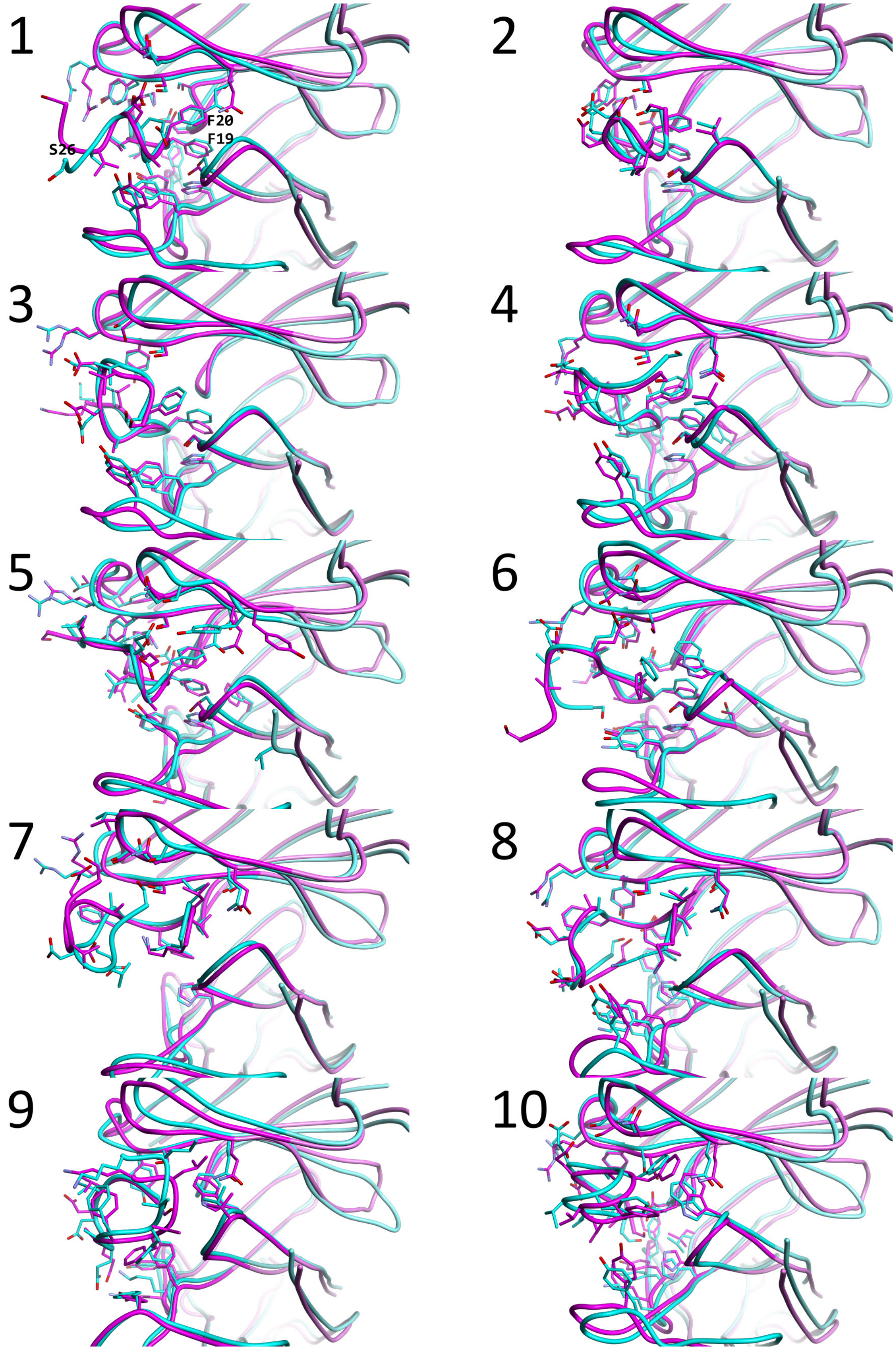

Figure S5. Refined structures $\mathbf{q}_k$ obtained from the canonical simulations starting from $\mathbf{r}_k$. Shown are the structures *k*=1-10 in cyan and magenta for $\mathbf{q}_k$ and $\mathbf{r}_k$, respectively. Also shown are the side-chains of solanezumab interacting with Aβ. Finally, for *k*=1, Phe19, Phe20 and Ser26 are indicated.

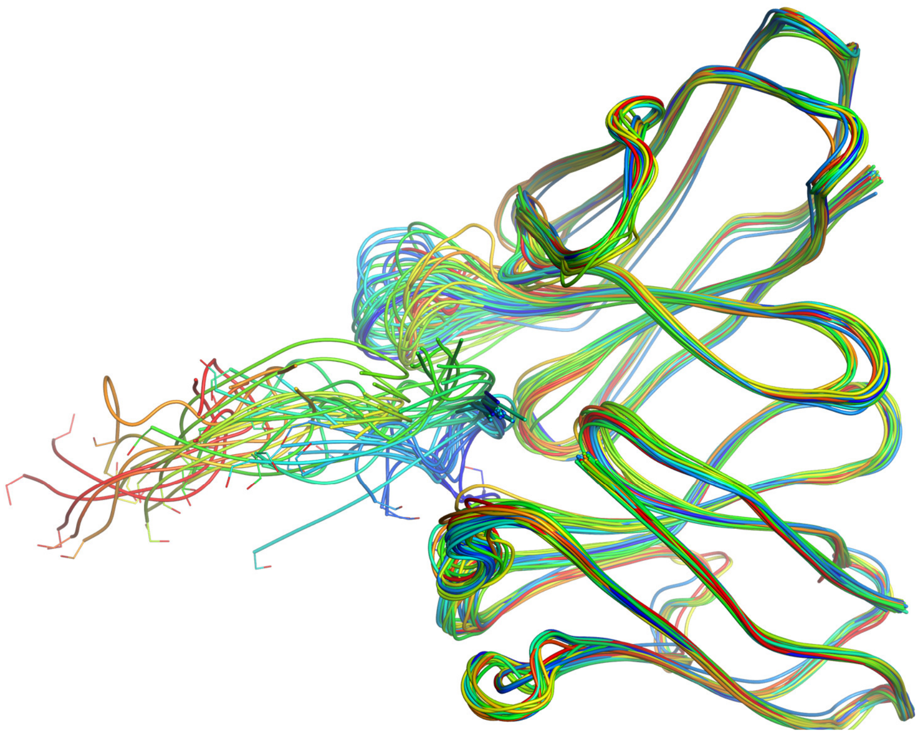

Figure S6. Initial structures that seeded the US simulations and were picked from the McMD ensemble. The structures are colored based on a blue-red gradient based on their window ID (see Table S2), where low λ′ values (bound) correspond to blue and high ones (unbound) to red.

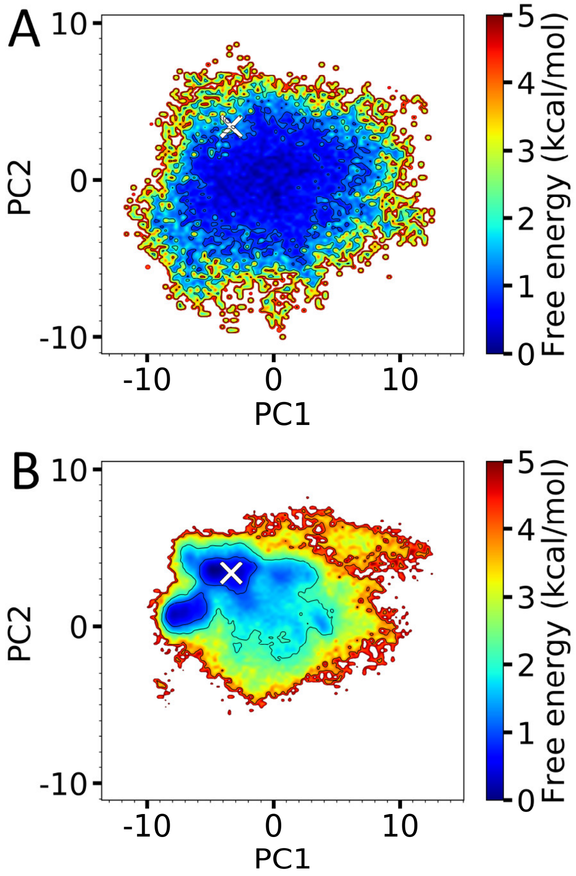

Figure S7. FEL obtained from the conformational ensemble of Aβ in isolation based on PCA with the distance matrix of all Cα pairs (excluding ± 3 neighboring Cα atoms). (*A*) FEL of Aβ in isolation at 300 K, with the experimental conformation indicated by the white cross. (*B*) FEL of Aβ in complex at 300 K, with the experimental conformation indicated by the white cross.

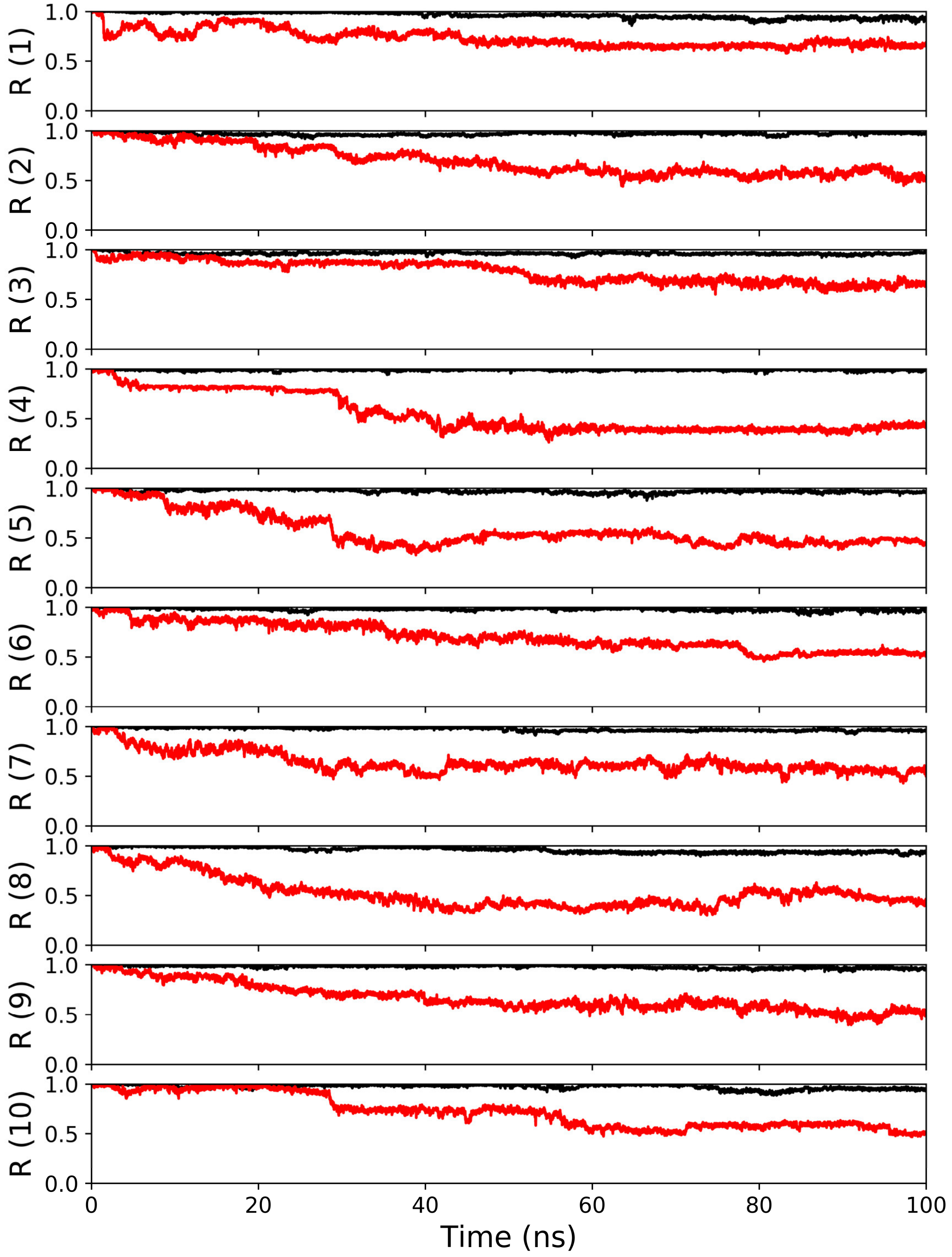

Figure S8. R-value plots of the first full-length model of Aβ produced from $\mathbf{r}_1$. Shown are the R-value plots of the canonical MD simulations at 300 K and 400 K in black and red, respectively, where 10 simulations were performed with different random seeds to initialize the velocities of each trajectory.

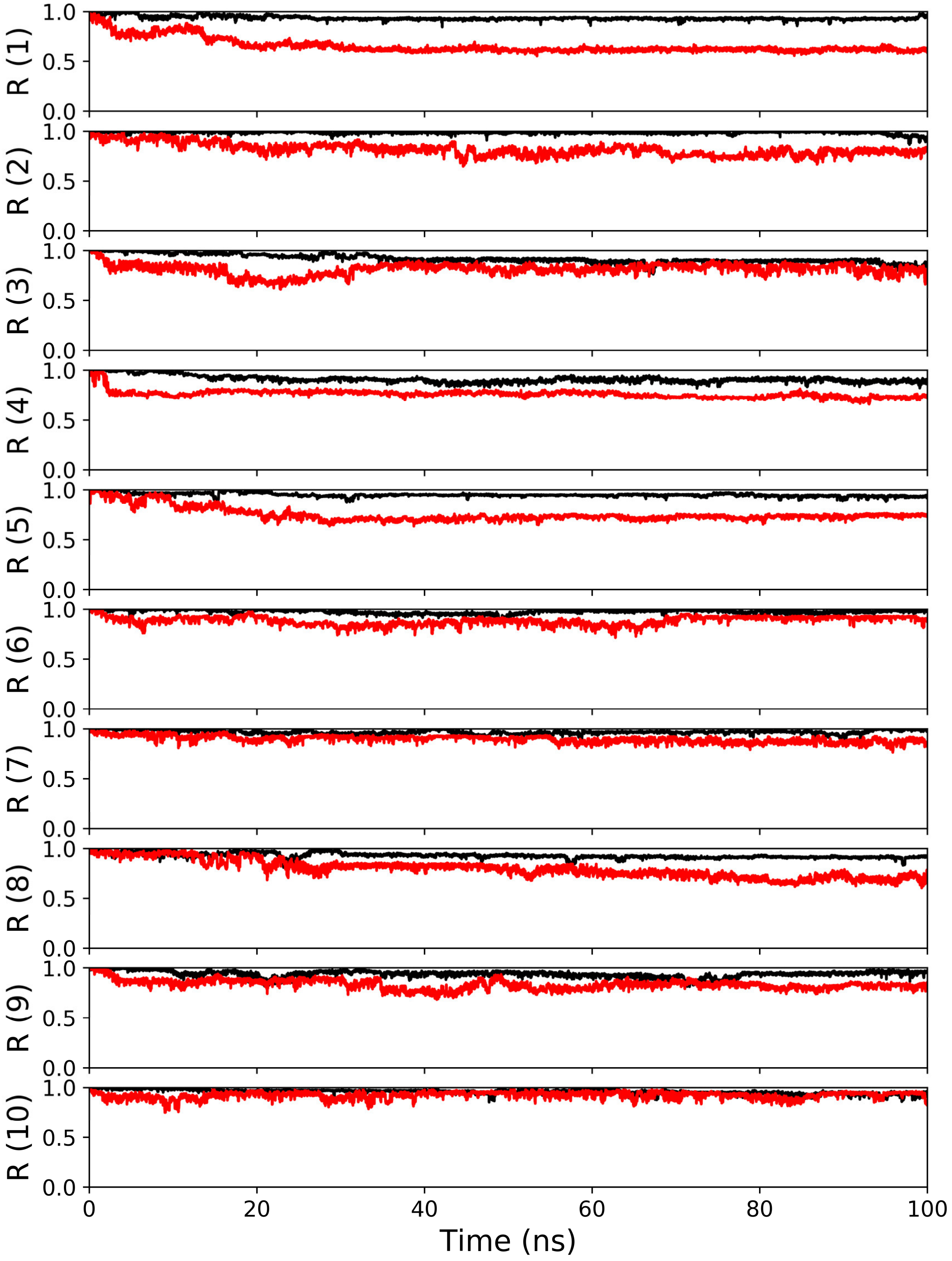

Figure S9. R-value plots of the second full-length model of Aβ produced from $\mathbf{r}_1$. Shown are the R-value plots of the canonical MD simulations at 300 K and 400 K in black and red, respectively, where 10 simulations were performed with different random seeds to initialize the velocities of each trajectory.

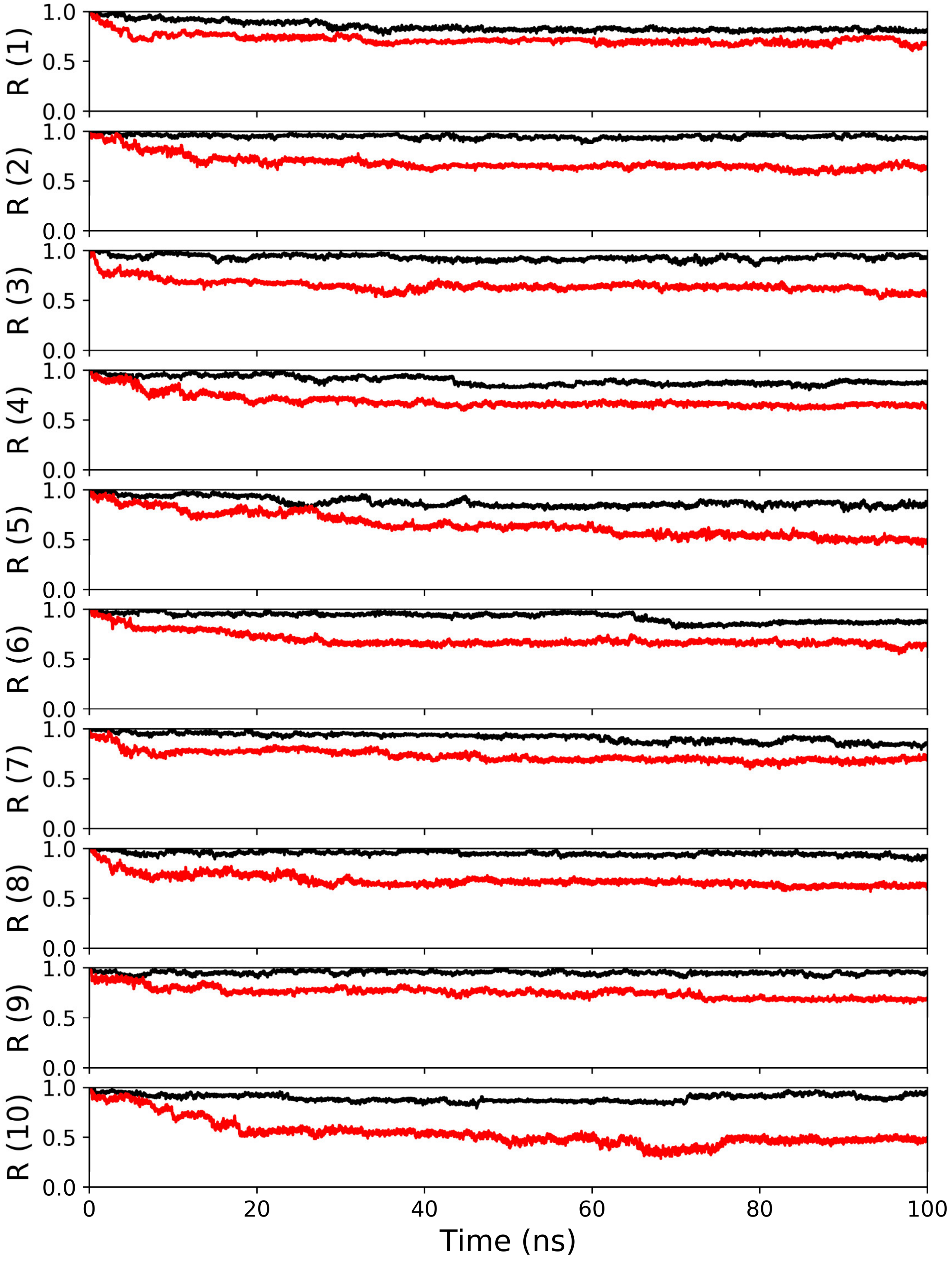

Figure S10. R-value plots of the first full-length model of Aβ produced from $\mathbf{r}_2$. Shown are the R-value plots of the canonical MD simulations at 300 K and 400 K in black and red, respectively, where 10 simulations were performed with different random seeds to initialize the velocities of each trajectory.

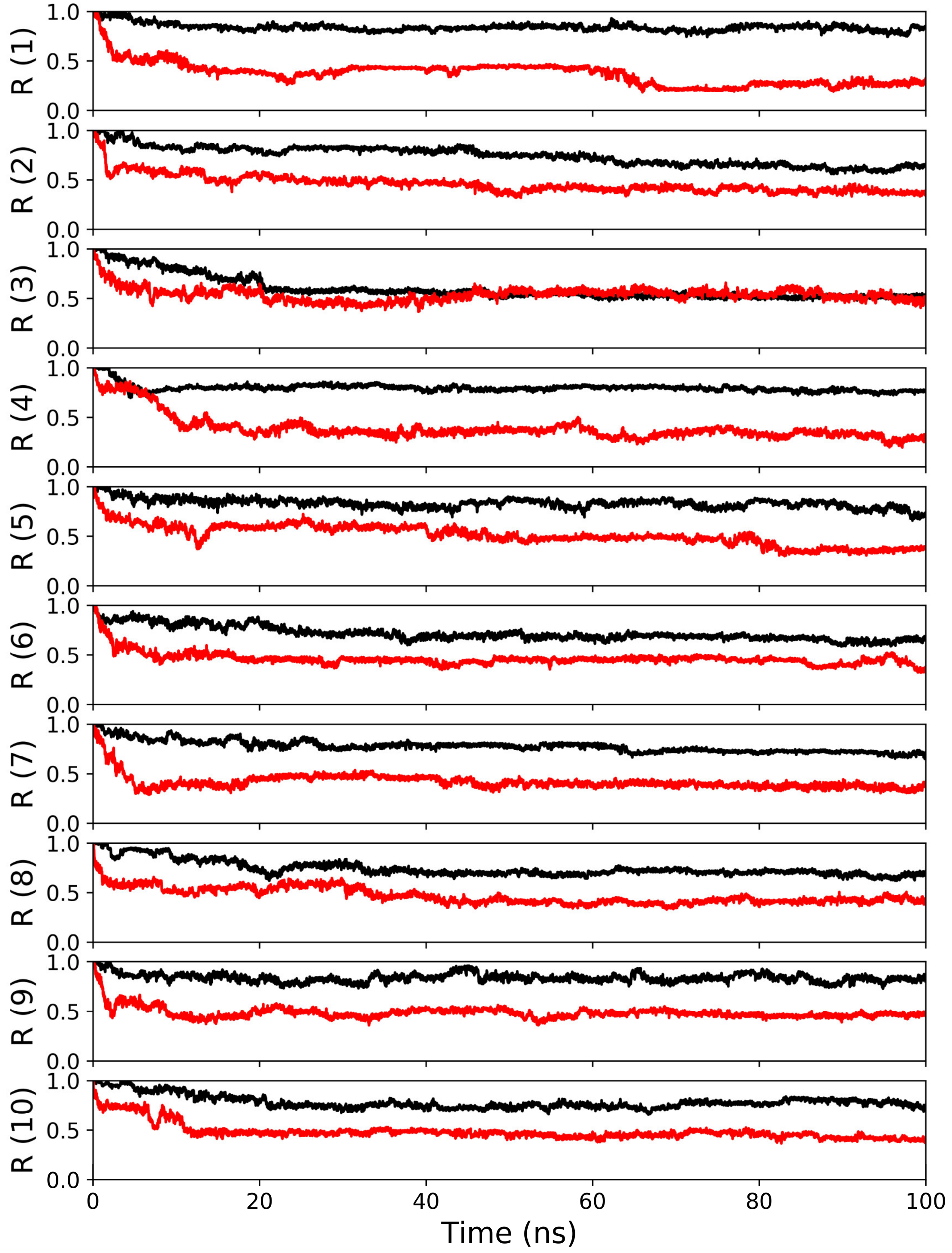

Figure S11. R-value plots of the second full-length model of Aβ produced from $\mathbf{r}_2$. Shown are the R-value plots of the canonical MD simulations at 300 K and 400 K in black and red, respectively, where 10 simulations were performed with different random seeds to initialize the velocities of each trajectory.
